# Let’s stay in touch: Frequency (but not mode) of interaction between leaders and followers predicts better leadership outcomes

**DOI:** 10.1371/journal.pone.0279176

**Published:** 2022-12-22

**Authors:** Daniel Wroblewski, Annika Scholl, Lara Ditrich, Lotte Pummerer, Kai Sassenberg

**Affiliations:** 1 Social Processes Lab, Leibniz-Institut für Wissensmedien, Tuebingen, Germany; 2 University of Tuebingen, Tuebingen, Germany; Central China Normal University, CHINA

## Abstract

Successful leadership requires leaders to make their followers aware of expectations regarding the goals to achieve, norms to follow, and task responsibilities to take over. This awareness is often achieved through leader-follower communication. In times of economic globalization and digitalization, however, leader-follower communication has become both more digitalized (virtual, rather than face-to-face) and less frequent, making successful leader-follower-communication more challenging. The current research tested in four studies (three preregistered) whether digitalization and frequency of interaction predict task-related leadership success. In one cross-sectional (Study 1, *N* = 200), one longitudinal (Study 2, *N* = 305), and one quasi-experimental study (Study 3, *N* = 178), as predicted, a higher frequency (but not a lower level of digitalization) of leader-follower interactions predicted better task-related leadership outcomes (i.e., stronger goal clarity, norm clarity, and task responsibility among followers). Via mediation and a causal chain approach, Study 3 and Study 4 (*N* = 261) further targeted the mechanism; results showed that the relationship between (higher) interaction frequency and these outcomes is due to followers perceiving more opportunities to share work-related information with the leaders. These results improve our understanding of contextual factors contributing to leadership success in collaborations across hierarchies. They highlight that it is not the *digitalization* but rather the *frequency* of interacting with their leader that predicts whether followers gain clarity about the relevant goals and norms to follow and the task responsibilities to assume.

## Introduction

Across organizations and work settings, leaders’ primary objective is to influence their followers in a way that allows for reaching organizational goals [1–3]. Accordingly, successful leadership requires that leaders make followers aware of the goals, norms, and tasks that the former consider relevant [4–7]—so that followers know of what is expected from them, take responsibility, and engage on behalf of joint organizational success [8].

Social interactions between leaders and followers are key to reaching this awareness. Notably, the settings in which leaders and followers interact with one another and communicate about the goals or norms have been changing: Economic globalization and digitalization keep transforming work environments, with the result that leaders and followers work more frequently at different locations [9, 10]. On top of that, more flexible working hours, home office regulations, and, if nothing else, the COVID-19 pandemic have, on average, increased the (spatial) distance between leaders and followers across fields of work [11–14]. Since a virtual work setting exacerbates the control over leadership outcomes [15, 16], factors which ensure that employees know what to do and how to do it, as well as assume responsibility for their tasks in a virtual setting, are vital for organizational success.

The increasing virtuality of leader-follower interactions has two important implications: First, interactions between leaders and followers have become more *digitalized* (i.e., physically distant); they often take place virtually rather than face-to-face [10, 11, 17–19]. Second, leaders and followers likely experience opportunities for interaction less frequently; for instance, with leaders and followers working at different locations or at home, there are fewer chances of chit-chat after an official meeting or of incidentally meeting each other in the hallway [20]. Especially the latter aspect, lower *frequency* of interactions, may pose a challenge for leadership success: Due to less frequent contact, followers and leaders see fewer opportunities to share relevant work-related information with each other—which may ultimately make it harder for leaders to make the relevant goals to achieve, the norms to follow, and the task responsibilities to assume clear to their followers.

The present work focused on the role of *frequency* of interaction between followers and their leaders. It tested the hypothesis that more frequent interaction predicts better task-related leadership outcomes among followers (i.e., goal clarity, norm clarity, and task responsibility)—because more frequent contact provides more chances to share work-related information with each other. We also investigated the role of *digitalized* interaction and whether it predicts task-related leadership outcomes (beyond frequency).

Addressing these two facets of ‘distance’ between leaders and followers, the present work includes four studies on whether the frequency and the digital mode of contact between leaders and followers predict task-related leadership outcomes at a given moment (Study 1), over time (Study 2), and in a quasi-experimental study inducing different (high vs. low) levels of frequency of interaction (Study 3). In the last step, following a causal chain logic [21], we investigated how the assumed mediator—perceived opportunities for work-related information sharing between leader and follower—influences task-related outcomes in another quasi-experimental study (Study 4). In sum, the current research contributes to a better understanding of the preconditions for successful leadership in a globalized and digitalized work environment while targeting a concrete leadership context (i.e., having more or less frequent contact with followers as one aspect of leader-follower distance) as a potential means to contribute to better task-related leadership outcomes.

### How frequent interactions may contribute to leadership success

The frequency of leader-follower interaction reflects the “perceived degree to which leaders interact with their followers” [11 p686]. Accordingly, it refers to the *quantity* of contact—how often leaders and followers are in touch with each other. The frequency of interaction constitutes a specific aspect of a leadership setting that is considered theoretically independent of the *mode* of interaction, such as whether the interaction occurs digitally or face-to-face (the latter reflects ‘physical distance’) [11]; leaders can be physically distant (thus, interact with followers only indirectly via digital modes from a distant location) and yet have (more or less) frequent interactions with followers.

The frequency of interaction has been argued to constitute an important aspect that contributes to leadership outcomes (e.g., in times of increasing distance and digital contact [11]). But what exactly does the frequency of interaction *do*—that is, how does it affect followers? Some prior work targeted its role for *relational* outcomes. On a relational level, frequent interaction likely allows leaders and followers to exchange resources, effort, and emotional support [22], which contributes to high-quality relationships [23]. As such, more frequent interactions should help followers (and leaders) to establish better relations with each other [24–27]. Indeed, some evidence suggests that more frequent interaction is associated with better leader-follower relationships (e.g., more trust among followers in their leaders [28]) and better team mood [29].

Regarding the role of frequency of interaction for *task*-related leadership outcomes, several theoretical approaches suggest that communication has the potential to improve performance through better coordination, clarifying misunderstandings, and the exchange of information [30, 31]. Indeed, (curvilinear) relationships between communication *quality* and performance have been found for team interactions [32], as well as leader-follower interactions [33]. However, research regarding the link between *frequency* of leader-follower interaction and outcomes relevant to performance, such as goal clarity, norm clarity, and task responsibility, is scarce. As these factors are crucial for successful leadership [6, 7], a better understanding of how they arise is vital, particularly in a time where the number of teleworkers constantly rises.

The present work targets this gap precisely. Indeed, leaders and followers likely need opportunities to exchange work-related information with each other not only to establish a good interpersonal relationship; they likely also need frequent contact for task-related reasons—that is, to clarify *goals*, (i.e., followers’ solid understanding of what they are expected to strive for in a work context). Goal clarity contributes to team and organizational performance [34, 35]. This strongly emphasizes the importance of goal clarity among followers. We propose that more frequent interaction between leaders and followers should provide them with more opportunities to share work-related information with each other [11], such that leaders (and followers) get the chance to convey their expectations, pose or answer questions, and provide feedback and appreciation [10]—whereas less frequent interaction likely rather hinders followers from asking such questions and leaders from providing the relevant information and thus from clarifying potentially ambiguous goals. This idea is in line with prior work showing, in reverse, that when their roles and tasks are unclear, followers get in touch with leaders to ask for guidance [22]. Accordingly, we propose that more frequent interaction with leaders predicts more goal clarity among followers.

*Hypothesis 1*: The higher the frequency of interaction of followers with their leader, the higher will be followers’ goal clarity.

Leaders do not only guide followers regarding *what* they have to do (i.e., goals), but in many cases also regarding *how* they have to strive to reach the goals at hand—in other words about the norms they are expected to follow [36]. Clarity about these norms (sometimes also called process clarity) has, alongside goal clarity [8], been shown to be a predictor of team performance, as well as of job satisfaction [37]. In order to achieve norm clarity among followers, the frequency of leader-follower interaction might likewise be a crucial antecedent. Indeed, followers often infer which norms are relevant to the group. They may do so indirectly by observing leaders’ behavior (as potential role models [38]) or explicitly by receiving information from the leader [39–41]—both of which require at least some level of frequent contact. Accordingly, in addition to goal clarity, a more frequent exchange with the leader on work-related topics likely (explicitly or indirectly) provides followers with information about how things should be done at work, which should contribute to followers’ norm clarity. In line with this, related findings show that followers having the chance to observe their leader tend to mimic their leader’s behaviors [38, 42–45]. Building upon and going beyond these ideas, we propose that more frequent interaction with leaders predicts more norm clarity among followers.

*Hypothesis 2*: The higher the frequency of interaction of followers with their leader, the higher will be followers’ norm clarity.

Furthermore, through providing greater opportunities for work-related exchange, more frequent interactions with their leaders may give followers more chances to contribute at work (e.g., their own suggestions, opinions, or perspectives); perceiving these opportunities as followers may foster a sense of being more *involved* in task completion, work procedures, and relevant decisions [10]. These aspects ensure that employees are motivated to engage at work [2, 46, 47]. Indeed, empirical evidence suggests that sensing that they are more involved at work may also help followers recognize that their contributions are valuable and important for the leader, the team, and, ultimately, the organization [10]. In line with this notion, more frequent interaction may provide followers with opportunities to share and contribute work-related information—which should ultimately promote their engagement and, thus, their sense of responsibility for their work [48]. Accordingly, we assume that more frequent contact with their leader will add to a greater sense of *responsibility* among followers to take care of their tasks and to contribute their share to organizational success:

*Hypothesis 3*: The higher the frequency of interaction of followers with their leader, the higher will be followers’ perceived task responsibility.

As elaborated above, we assume that the predicted relations between frequency and these three task-related leadership outcomes (norm clarity, goal clarity, and task responsibility) result from followers’ perception of having greater *opportunities to share* work-related information with their leader: more frequent interactions with their leader should give followers the impression that they can ask questions and make contributions; in contrast, less frequent contact may set the perceived ‘bar’ for getting in touch with the leader and asking questions if something is unclear much higher. Via more opportunities for work-related information sharing, followers likely attain more explicit clarity regarding what is expected from them (norms) and which end states to achieve (goals). Beyond that, sharing work-related information may also give followers a sense of being included in work procedures and that their contributions are important [10], thus promoting their sense of responsibility for their tasks (task responsibility). These notions are also in line with the communication literature, positing processes like the exchange of information as a means to improve performance [30–32]. Taken together, this results in the following mediation hypothesis:

*Hypothesis 4*: The relations between higher frequency of interaction and the task-related leadership outcomes are mediated by more perceived opportunities for work-related information sharing between leader and follower.

### The role of digitalized interaction for task-related leadership outcomes

With higher spatial distance, leader-follower interactions almost naturally become more digitalized. Digitalization here reflects the relative amount of digital (rather than face-to-face) contact between leaders and followers and has been argued to have different consequences.

On the one hand, some approaches assume that collaborating over (spatial) distance with a lack of face-to-face contact and (often) asynchronous modes of virtual communication—be it via text-, audio-, or video-based communication channels—makes it more *challenging* for leaders to manage collaboration with followers and motivate them towards completing their tasks [12, 16, 49, 50]. This might be attributable to the richness of information that is transferred via digital means. According to media-richness theory [51, 52], different modes of interaction (e.g., digital vs. face-to-face) hold a different richness of information, face-to-face being the richest, due to the number of social cues (e.g., facial expressions, gestures) that are transmitted during the interaction. As per media-richness-theory [51, 52], a reduction of this information richness leads to an increase of the equivocality of the information given. In terms of goals, norms, and tasks, this might prove problematic, since a greater equivocality may hinder followers from gaining a clear understanding of the goals and norms they are expected to follow, as well as from assuming responsibility for their tasks. Other approaches, however, argue that when leaders invest extra effort [50], learn how to use virtual modes of communication, or use media to augment leadership [49, 53], these challenges may be mitigated. Moreover, over the years, the approach towards media richness has shifted from an objective to a rather subjective view, meaning that the perceived richness of a mode of interaction rather depends, for instance, on attitudes towards, experience with, and knowledge about the medium (for a review, see [54]). According to this, a medium that may have very poor information richness to one individual, due to lacking experience with the former, might constitute a medium that is very rich on the information transferred for another person [54]. As per this notion, given that digital contact nowadays is part and parcel of everyday work in many occupations, leaders and followers gain more and more experience with digital communication; accordingly, the challenges and advantages of digital contact may eventually balance each other out, implying that despite a digital mode of interaction, followers may obtain a clear picture of the goals, norms, and tasks expected of them to follow and assume. This assumption is in line with approaches to computer-mediated communication, showing that interpersonal and task-related outcomes between digital versus face-to-face interaction differ only at the very beginning of an interaction (e.g., in one-shot interaction), but diminish soon over time [55]. Therefore, we believe that the following hypothesis suggested by early approaches to digitalization deserves to be retested because it might nowadays not be valid anymore.

*Hypothesis 5*: Digitalization of interaction of followers with their leader predicts less goal clarity, norm clarity, and less task responsibility among followers.

### The current work

**Fig 1:**
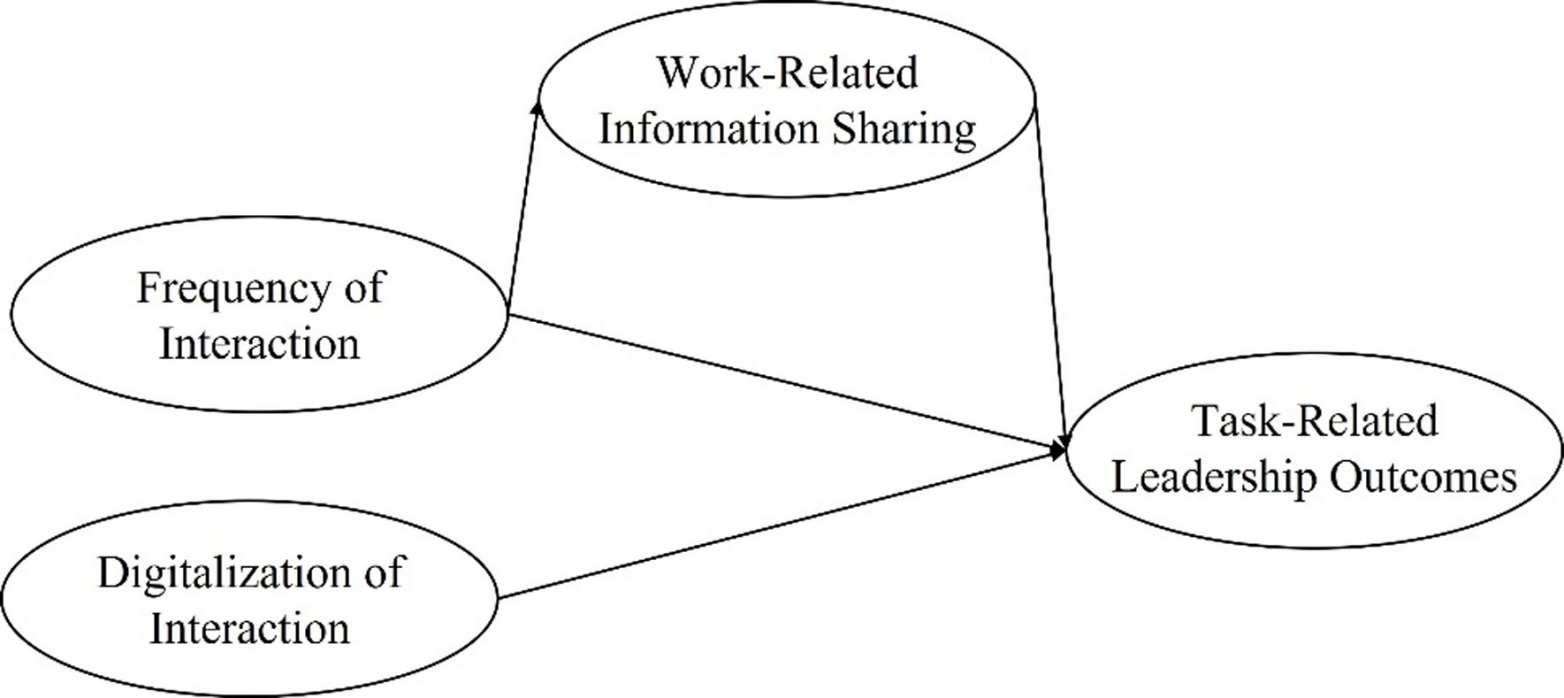
Theoretical model tested in the present research. Task-related leadership outcomes here are: norm clarity, goal clarity, and perceived task responsibility.

The present work tested the role of frequency and digitalization of interaction between leaders and followers for task-related leadership outcomes. The entire proposed model is displayed in Fig 1. Specifically, we predicted that the higher the frequency of interaction of followers with their leader, the higher will be (1) followers’ goal clarity (Hypothesis 1), (2) followers’ norm clarity (Hypothesis 2), and followers’ perceived task responsibility (Hypothesis 3). Furthermore, we expected that these relations are mediated by more perceived opportunities for work-related information sharing (Hypothesis 4). Additionally, we predicted that the digitalization of interaction between leaders and followers predicts less follower goal clarity, norm clarity, and perceived task responsibility (Hypothesis 5).

To provide high external validity, we first tested these hypotheses in two studies in the field with two independent samples of followers across organizations. Study 1 comprised a cross-sectional design as a first test of the predictions; Study 2 complemented this test with a longitudinal design with a time lag of six months between two measurement points. In doing so, Studies 1 and 2 assessed the frequency and the digitalization of interaction as predictors (Hypotheses 1, 2, 3, & 5). As outcomes across all studies, we assessed followers’ goal clarity, norm clarity, and task responsibility.

Going beyond and to allow for greater internal validity, we then ran two quasi-experimental studies that asked employees to recall real past work situations. Specifically, Study 3 took a quasi-experimental approach of asking followers to recall a time with a high (vs. low) frequency of contact with their leader (to compare different levels of frequency) before assessing task-related leadership outcomes. Finally, Study 4 followed a causal chain logic [21] by comparing high vs. low levels of the presumed mediator (opportunities for work-related information sharing) of Hypotheses 1–3; here, followers recalled a situation in which all their contact with their leader had included high (vs. low) levels of opportunities to exchange work-related information before assessing the three outcomes.

## Study 1: Initial test in the field

As an initial test of our predictions, we implemented a cross-sectional design via an online study among followers. We assessed frequency of interaction and digitalization of interaction and tested how they related to our three task-related leadership outcomes (goal clarity, norm clarity, and perceived task responsibility).

### Method

#### Transparency and openness

Ethical approval for the procedure of the current study was obtained by the Ethics Committee of the Leibniz-Institut für Wissensmedien under ‘LEK 2021/141’. Our sampling plan, data exclusions, and all measures are described in the study (see table in S2 Table for a full list of measures and table in S3 Table for a comprehensive list of items). The data and research materials of Study 1 are available at https://doi.org/10.23668/psycharchives.12200, the associated analysis code is available at https://doi.org/10.23668/psycharchives.12201. Data were analyzed using SPSS, version 25, and Mplus 8.5 [56]. The study, including its hypotheses and analyses, was preregistered at https://aspredicted.org/c4bn5.pdf.

#### Design and participants

The study was cross-sectional. Participants (personal contacts, i.e., employees across a range of businesses in Germany) were approached personally via email. An a-priori power-analysis (G*Power [57]) for 1–β = .80, α = .05, and *f*^2^ = 0.05 (small-to-medium effect) with two variables as predictors in a multiple regression analysis indicated an ideal sample size of *N* = 196. Preregistered inclusion criteria for participants were (1) being at least 18 years old, (2) having current continuous employment to the extent of at least 50% over the past six months, (3) having one or more direct leader(s), (4) answering the survey until the last item (in the exploratory measure interaction valence), and (5) no withdrawal of consent to use their data at the very end of the study (after debriefing).

We recruited 243 respondents to compensate for potential dropouts and exclusions in February and March 2021. Written informed consent was obtained at the beginning of the study. Six participants did not give consent in the beginning, one withdrew their data after the study, 30 did not finish the survey, and six participants did not work at least part-time over the last six months. Accordingly, data from the final sample of *N* = 200 participants (78 female, 122 male, *M*_age_ = 40.53, range = 20–63) were analyzed. Participants worked on average 40.01 hours (*SD* = 9.07) per week. The majority were employed in industry, consumption, and technology (76%); the other occupational fields were health, social services and education (7%), finance, consulting and insurance (5.5%), and other industries (11.5%). At the time of the survey, 75.5% of the participants had been working for their employers for more than five years.

#### Procedure

Employees were invited to participate in a 10-minute online survey on the “perception of aspects of work”. Participants first indicated whether they fulfilled the criteria for participation; then, we measured the predictors frequency of interaction and digitalization of interaction, both regarding participants’ interaction with their leader over the last six months. Next, we measured the task-related leadership outcomes goal clarity, norm clarity, and perceived task responsibility, and two control variables (i.e., perceived appropriateness of frequency of interaction and interaction valence). Finally, participants answered demographic questions (gender, age, working hours, and their area of business).

#### Measures

Unless indicated otherwise, all items were rated on a seven-point scale ranging from 1 =“strongly disagree” to 7 =“strongly agree”.

*Predictors*. *Frequency of interaction* was measured with four items (e.g., “How frequently did you interact with your leader at work?” [58]; 7-point scale ranging from 1 = “Once or twice in six months” to 7 = “Many times daily”). We assessed *digitalization of interaction* with one item (“What percentage of your weekly working time did you work away from your office (e.g., home-office)?”).

*Goal clarity*. Four items assessed the dependent variable *goal clarity* (e.g., “In my job, most goals are reasonably clear.”). For this scale, the wording of the original items from Burman and colleagues [59] was tailored to the current research (e.g., original label: “In all domains, goals are poorly described and/or lack relevance for fellowship training and career development” was turned into “In my job, goals are poorly described.”).

*Norm clarity*. *Norm clarity* as dependent variable was measured via eight items (e.g., “In my team, we agree about how things should be done.”). The scale was self-generated as, to our knowledge, there was no established measure for this concept.

*Perceived task responsibility*. As a third dependent variable, we included five items as indicators of *perceived task responsibility*, adapted from Schaumberg and Flynn [60]. Again, the items’ original wording was tailored to the current research (e.g., original label “I feel a great deal of responsibility for the people I work with” was turned into “In general, I feel a great deal of responsibility for the tasks related to my work.”).

### Results

Cronbach’s α, descriptive statistics, and correlations are displayed in Table 1. We conducted three separate multiple regression analyses to test our hypotheses that frequency of interaction and digitalization of interaction (added simultaneously as predictors) predict (a) goal clarity, (b) norm clarity, and (c) perceived task responsibility, respectively.

**Table 1:** Means, standard deviations, correlations (Pearson’s r), and Cronbach’s α (in brackets on the diagonal) of Study 1 (*N* = 200), Study 3 (*N* = 178) and Study 4 (*N* = 261).

***Study 1***							
Variables	*M*	*SD*	(1)	(2)	(3)	(4)	(5)
(1) Frequency	4.39	1.17	(.86)				
(2) Digitalization	82.00	26.14	-.15*	(-)			
(3) Goal clarity	4.71	1.41	.25***	-.01	(.84)		
(4) Norm clarity	4.64	0.87	.17*	.03	.51***	(.75)	
(5) Responsibility	6.06	0.68	-.02	.05	.11	.15*	(.62)
***Study 3***							
Variables	*M*	*SD*	(1)	(2)	(3)	(4)	(5)
(1) Goal clarity	5.04	1.27	(.79)				
(2) Norm clarity	5.32	1.13	.57***	(.74)			
(3) Responsibility	5.70	0.87	.30***	.42***	(.73)		
(4) Digitalization	4.37	1.96	.12	-.05	.02	(.92)	
(5) Info sharing	5.31	1.49	.58***	.55***	.39***	-.17*	(.91)
***Study 4***							
Variables	*M*	*SD*	(1)	(2)	(3)	(4)	(5)
(1) Goal clarity	5.04	1.27	(.84)				
(2) Norm clarity	5.32	1.13	.63***	(.81)			
(3) Responsibility	5.70	0.87	.33***	.43***	(.81)		
(4) Frequency	4.37	1.96	.25***	.25***	.17**	(.81)	
(5) Digitalization	5.31	1.49	.03	-.02	-.04	-.34***	(.89)

*p < .05.

** *p* < .01.

*** *p* < .001.

Regression models are reported in Table 2. Results indicated that a higher *frequency* of interaction predicted more goal clarity (*b* = 0.30, *SE* = 0.09, *p* < .001) and norm clarity (*b* = 0.13, *SE* = 0.05, *p* = .013), but not more perceived task responsibility (*b* = -0.01, *SE* = 0.04, *p* = .805). Accordingly, we found support for H1 and H2, but not for H3. *Digitalization* of interaction neither predicted goal clarity (*b* = 0.00, *SE* = 0.00, *p* = .727), nor norm clarity (*b* = 0.00, *SE* = 0.00, *p* = .457), nor perceived task responsibility (*b* = 0.00, *SE* = 0.00, *p* = .534), thus not supporting H5. In sum, the frequency of interaction (but not digitalization of interaction) between followers and their leaders predicted better leadership outcomes in terms of goal clarity and norm clarity.

**Table 2:** Multiple regression analyses of Study 1 (*N* = 200).

	Goal clarity				Norm clarity				Responsibility			
	Model	*b*	*SE*	*p*	Model	*b*	*SE*	*p*	Model	*b*	*SE*	*p*
Frequency		0.30	0.09	< .001		0.13	0.05	.013		-0.01	0.04	.805
Digitalization		0.00	0.00	.727		0.00	0.00	.745		0.00	0.00	.534
*R* ^*2*^	0.06				0.03				0.00			
*R* ^*2*^_*adj*_	0.05				0.02				-0.01			
*F*(2, 197)	6.49				3.25				0.25			

**Fig 2:**
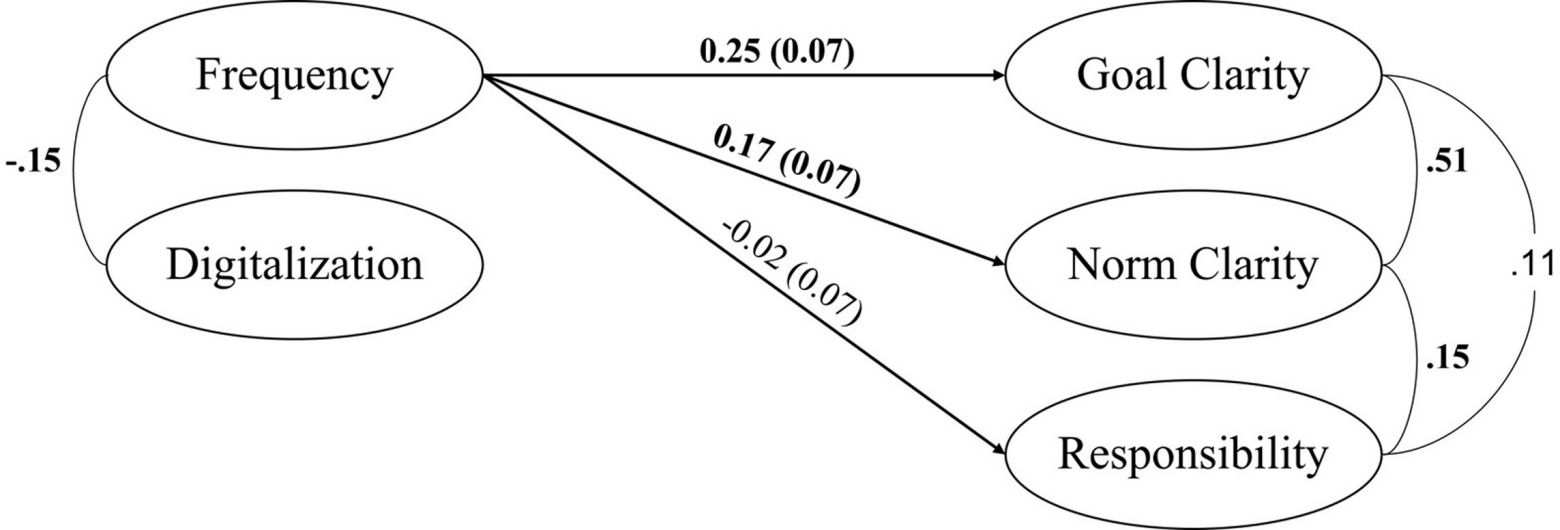
Structural model tested in Study 1 including H1–H4 (*N* = 200). Estimates in bold are significant.

To capture all predicted paths in one model, we also applied structural equation modeling (SEM) using Mplus 8.5 [56] to test all hypotheses at once (for structural model, see Fig 2). Since we had not found a relation between digitalization of interaction and the task-related leadership outcomes, we set the paths from digitalization to the outcomes equal to zero. The results indicated a good model fit, χ^2^(3) = 0.83, *p* = .842, CFI = 1.000, RMSEA = .000, 90% CI [.000;.067], SRMR = .019. Frequency of interaction once again predicted more goal clarity (*b* = 0.25, *SE* = 0.07, *p* < .001) and more norm clarity (*b* = 0.17, *SE* = 0.07, *p* = .017), but not more task responsibility (*b* = -0.02, *SE* = 0.07, *p* = .727, STDXY standardization, from Mplus). As such, results of the SEM once again supported H1 and H2—but not H3. Given that model fit was high and the path from digitalization to the outcomes was fixed to zero, the model can also be seen as indirectly contradicting H5.

### Discussion

Study 1 yielded first correlational evidence that frequency of interaction is linked to better task-related leadership outcomes in terms of more goal clarity and norm clarity. In contrast, beyond frequency, the digitalization of interaction was not linked to any of the leadership outcomes examined here. Given that both Hernandez and colleagues [29] and Shockley and colleagues [33] found a curvilinear relation between frequency of interaction and socio-emotional outcomes, we tested for such a pattern. However, we did not find any evidence for it, indicating that the relation between frequency of interaction and the outcomes is in fact linear. Accordingly, our results yield first evidence in line with H1 and H2, but not H3 (perceived task responsibility) or H5.

A potential reason for the lack of support for H3 may be that the mean of perceived task responsibility was rather high, while the variability was very small (*M* = 6.06, *SD* = 0.68), which may have produced a ceiling effect. The lack of support for H5 is in line with the critical discussion of this prediction in our introduction. However, it should be noted that our self-generated one-item measure for digitalization was not optimally suited to test the prediction. Another clear limitation is that the results here rely on cross-sectional data. To address these limitations, Study 2 used a longitudinal design and an improved measure of digitalization to test whether the frequency of interaction and the digitalization of interaction are linked to leadership outcomes at a later point in time.

## Study 2: Longitudinal study

This study examined whether frequency and digitalization of interaction at one point in time are linked to task-related leadership outcomes six months later.

### Method

#### Transparency and openness

Ethical approval for the procedure of the current study was obtained by the Ethics Committee of the Leibniz-Institut für Wissensmedien under ‘LEK 2021/141’. We again describe our sampling plan, data exclusions, and all measures in the study (see table in S4 Table for a full list of measures and table in S5 Table for a comprehensive list of items). The data and research materials of Study 2 are available at https://doi.org/10.23668/psycharchives.12200, the associated analysis code is available at https://doi.org/10.23668/psycharchives.12201. Data were analyzed using SPSS, Version 25, and Mplus 8.5 [56]. This study’s design and analyses were not preregistered (for details, see below and see text in S3 Text).

#### Design and participants

The study comprised a longitudinal design with two measurement points. Four hundred and twenty-three people were recruited through Prolific Academic at Time 1 (t1; September 2019, in the U.K.) for a two-wave online study on “work perception”. Written informed consent was obtained at the beginning of the study. Five of them did not give consent in the beginning, 22 withdrew their data after the study, four had no direct supervisor, and one participant indicated not having worked at all (the latter two refer to preregistered criteria). Accordingly, data from *N* = 391 participants (233 female, 156 male, 1 other, and 1 missing, *M*_age_ = 37.96, range = 19–64) were included at t1. Participants at t1 worked on average 35.88 hours (*SD* = 9.30) per week. The majority were employed in healthcare, social services, and education (33.2%); the other occupational fields were industry, consumption, and technology (26.9%), finance, consulting, and insurance (6.9%), and other industries (33.0%). At the time of the survey, 45.5% of the participants had been working for their employers for less than five years.

For Time 2 (t2, March 2020), we implemented a time lag of six months—we expected that the examined variables need a certain period of time to develop, since goals and norms are relatively stable. Again, we obtained written informed consent at the beginning of the survey. At t2, 344 of the previously recruited participants completed the questionnaire once again; out of these, 37 were excluded due to a job change between t1 and t2. Two additional participants had to be excluded because an error in their Prolific ID led to difficulties in matching the respective datasets of t1 and t2. Thus, data from 305 participants (176 female, 125 male, one other, and three missing, *M*_*age*_ = 39.03, range = 19–65) were included at t2. Accordingly, we analyzed combined data from *N* = 305 participants at t1 and t2 together, which is reported here. At t2, 100% of participants indicated working for the same employer as at t1. Average working hours were indicated with 35.51 hours (*SD* = 9.47). Most participants were employed in healthcare, social, and education (32.5%); the remainder was employed in industry, consumption, and technology (22.0%), finance, consulting, and insurance (8.9%), and other industries (36.7%). At t2, 39.7% of the participants had been working for their employers for less than five years (*M* = 2.14, *SD* = 1.24).

Note that as the study was initially planned for another purpose (for details, see text in S3 Text), the sample size was determined by a power analysis not tailored to the current research question. Therefore, we report a sensitivity analysis rather than an a priori power analysis. The sensitivity analysis (G*power [57]) revealed that our actual sample (*N* = 391) was large enough to detect effects of *f*^*2*^ = 0.02 with α = .05 and 1-β = .80 (for a single regression weight in a multiple regression with two predictors). Preregistered inclusion criteria (for the study’s initial purpose) for participants were once again (1) having a leader (in terms of a direct supervisor), (2) working at least part-time, and (3) not withdrawing their data after the debriefing for the study. Referring to (2), we originally planned only to include participants working at least 18 hours/week. However, as we found that including participants who worked at all did not change the results, we included all participants working more than 0 hours/week.

#### Measures

Unless indicated otherwise, all items used a seven-point scale ranging from 1 =“strongly disagree” to 7 =“strongly agree”. All measures were assessed both at t1 and t2 (to allow for testing the full model, including ‘reverse causality’).

*Predictors*. We measured *frequency of interaction* with a four-item scale at t1 and t2 (e.g., “In general, I interact with my leader often.”). We here chose items that were clearly unrelated to whether the interaction with the leader was initiated by the leader or the follower (as compared to Study 1).

We assessed *digitalization of interaction* with a more comprehensive three-item scale (e.g., “My leader and I mostly interact using media (e.g., telephone, email) rather than face-to-face.”) instead of using a single item. The third item, which measured the proportion of virtual contact on a scale from 0 to 100%, was converted to a 7-point scale before combining it with the other two items into one scale. This improvement of the measure should lead to a higher chance of finding a possible effect of digitalization of interaction on our outcomes, compared to Study 1.

*Outcomes*. Goal clarity, norm clarity, and perceived task responsibility were measured with the same scales as in Study 1.

### Results

Descriptive statistics, correlations, and internal consistencies (α) are reported in Table 3. Again, we tested our hypotheses via three multiple regression analyses that examined how frequency of interaction and digitalization of interaction, both at t1 (again, entered simultaneously into the regression model), are linked to the three leadership outcomes goal clarity, norm clarity, and perceived task responsibility at t2, while controlling for the respective leadership outcome at t1.

**Table 3:** Means, standard deviations, correlations (Pearson’s r), and Cronbach’s α (in brackets) on the diagonal of Study 2 (*N* = 305).

*Variables*	*M*	*SD*	(1)	(2)	(3)	(4)	(5)	(6)	(7)	(8)	(9)	(10)
Time 1												
(1) Frequency	5.48	1.14	(.79)									
(2) Digitalization	2.72	1.38	-.39***	(.67)								
(3) Norm clarity	5.15	0.83	.29***	-.14**	(.81)							
(4) Goal clarity	4.72	1.36	.29***	-.08	.58***	(.86)						
(5) Responsibility	5.44	1.01	.22***	.02	.35***	.36***	(.81)					
Time 2												
(6) Frequency	5.56	0.96	.71***	-.36***	.30***	.22***	.26***	(.71)				
(7) Digitalization	2.99	1.48	-.31***	.70***	-.14*	-.03	-.02	-.36***	(.70)			
(8) Norm clarity	5.17	0.86	.34***	-.17**	.64***	.41***	.24***	.37***	-.07	(.83)		
(9) Goal clarity	4.86	1.33	.32***	-.10	.50***	.66***	.31***	.24***	-.02	.61***	(.87)	
(10) Responsibility	5.47	0.98	.30***	-.02	.31***	.27***	.69***	.27***	.08	.40***	.36***	(.82)

* *p* < .05.

** *p* < .01.

*** *p* < .001.

In line with our hypotheses and results from Study 1, a higher frequency of interaction at t1 was linked to more goal clarity at t2 (*b* = 0.15, *SE* = 0.06, *p* = .013), more norm clarity at t2 (*b* = 0.11, *SE* = 0.04, *p* = .008), and more perceived task responsibility at t2 (*b* = 0.12, *SE* = 0.04, *p* = .006). Digitalization of interaction at t1, however, neither predicted goal clarity (*b* = 0.00, *SE* = 0.05, *p* = .924), nor norm clarity (*b* = 0.00, *SE* = 0.03, *p* = .951), nor perceived task responsibility (*b* = 0.04, *SE* = 0.03, *p* = .218) at t2, again contradicting H5. All models are reported in Table 4.

**Table 4:** Multiple regression analyses of Study 2 testing how digitalization and frequency of interaction (both t1) predict leadership outcomes (t2; *N* = 305).

	Goal clarity					Norm clarity					Task Responsibility				
Predictor	*R* ^*2*^ *adj*	*F*	*b*	*SE*	*p*	*R* ^*2*^ *adj*	*F*	*b*	*SE*	*p*	*R* ^*2*^ *adj*	*F*	*b*	*SE*	*p*
**Model 1**	0.43	234.04			< .001	0.41	212.41			< .001	0.48	281.37			< .001
Outcome (t1)			0.65	0.04	< .001			0.69	0.05	< .001			0.68	0.04	< .001
**Model 2**	0.44	81.82			< .001	0.42	75.05			< .001	0.49	98.06			< .001
Outcome (t1)			0.61	0.04	< .001			0.64	0.05	< .001			0.65	0.04	< .001
Frequency			0.15	0.06	.013			0.11	0.04	.008			0.12	0.04	.006
Digitalization			0.00	0.05	.924			0.00	0.03	.951			0.04	0.03	.218

For outcome goal clarity t2, ‘outcome t1’ refers to goal clarity t1; for outcome norm clarity t2, ‘outcome t1’ refers to norm clarity t1; for outcome task responsibility t2, ‘outcome t1’ refers to task responsibility t1.

Subsequently, we again implemented a structural equation model using Mplus 8.5 [56] that captured all hypotheses. We estimated the structural model with leader distance at t1 predicting leadership outcomes at t2, while controlling for leadership outcomes at t1. This model also included the reverse direction (leadership outcomes at t1 predicting frequency at t2 while controlling for frequency at t1). We set the correlations between digitalization and the leadership outcomes equal to zero, representing the null effects we anticipated based on the results of Study 1. The results of the structural model are presented in Table 5.

**Table 5:** Estimates of the structural model in Study 2, with *b* (*SE*); STDXY standardization from Mplus (*N* = 305).

	Time 2			
*Variables*	Goal clarity	Norm clarity	Responsibility	Frequency
**Time 1**				
**Frequency**	0.21 (0.05)***	0.19 (0.05)***	0.16 (0.05)**	0.43 (0.07)***
**Digitalization**	0.00 (0.00)	0.00 (0.00)	0.00 (0.00)	-
Goal clarity	0.44 (0.05)***	-	-	-0.09 (0.05)
Norm clarity	-	0.40 (0.05)***	^-^	0.08 (0.06)
Responsibility	-	-	0.50 (0.05)***	0.10 (0.06)

Hypothesized paths are highlighted in bold.

* *p* < .05.

** *p* < .01.

*** *p* < .001.

The findings indicated a good model fit, χ^2^(10) = 11.36, *p* = .330, CFI = .998, RMSEA = .021, 90% CI [.000;.066], SRMR = .029. As hypothesized and mirroring findings from the separate regressions, frequency of interaction (t1) was linked to more goal clarity (*b* = 0.21, *SE* = 0.05, *p* < .001), norm clarity (*b* = 0.19, *SE* = 0.05, *p* < .001), and perceived task responsibility (*b* = 0.16, *SE* = 0.05, *p* = .002) all at t2 (STDXY standardization, from Mplus). This supports H1–H3 that the frequency of interaction between leaders and their subordinates at t1 (but not the digitalization of interaction at t1, contrary to H5) links to better leadership outcomes at t2.

### Discussion

Study 2 once again supported our hypotheses that frequency of interaction links to better leadership outcomes in terms of goal clarity, norm clarity, and (in contrast to Study 1) perceived task responsibility. This clearly extends the findings from Study 1, supporting the predicted relations over time. Notably, the relation with perceived task responsibility (H3) had not been supported in Study 1 but was found in Study 2. This might be attributable to the lower levels of responsibility reported by participants in Study 2 (*M* = 5.44 for t1, *M* = 5.47 for t2). Consequently, the discussed ceiling effect may not have been present here, which may have better allowed the predicted relation to manifest. Again, digitalization of interaction did not predict any of the leadership outcomes, replicating the results of Study 1. Accordingly, H5 was again not supported.

As indicated earlier, Study 2 was originally conducted to test a different set of predictions. In this sense, the reported analyses are exploratory. Notwithstanding, the results do replicate the findings of Study 1 (except for H3) and provide support for the predictions preregistered for that study. While the results of Studies 1–2 provide high external validity in different work contexts, they also relied on correlational data. To overcome this limitation, Study 3 used a quasi-experimental design in which we induced different levels of frequency of interaction.

## Study 3: Comparing high versus low frequency

In this study, we quasi-experimentally induced and compared a high vs. low frequency of interaction between followers and their leader (controlling for digitalization of interaction) and tested the effects on followers’ goal clarity, norm clarity, and perceived task responsibility. Given that the results of both Studies 1 and 2 did not show any effect of digitalization on the outcomes, we did not target it as a predictor in this study. However, we did not want to exclude it entirely, which is why digitalization was assessed as a control variable (i.e., entered in all main analyses for the sake of consistency, following the procedure from Studies 1–2 which had included both frequency and digitalization as predictors).

### Method

#### Transparency and openness

Approval for this study was obtained by the Ethics Committee of the Leibniz-Institut für Wissensmedien under ‘LEK 2021/141’. The sampling plan, all data exclusions, and all measures are described in the study (see table in S6 Table for a full list of measures and table in S7 Table for a comprehensive list of items). The data and research materials of Study 3 are available at https://doi.org/10.23668/psycharchives.12200, the associated analysis code is available at https://doi.org/10.23668/psycharchives.12201. We analyzed the data using SPSS, version 25, and PROCESS (version 3.5 [61]). The study, including its hypotheses and analyses, was preregistered (https://aspredicted.org/eh8vd.pdf).

#### Design and participants

As in Study 1 and 2, this study again investigated whether frequency of interaction predicts task-related leadership outcomes. However, instead of a cross-sectional or longitudinal design, the study featured a quasi-experimental design, implementing two between-participants conditions: by asking participants to remember a time in which they had frequent (vs. infrequent) contact with their leader, we incorporated one condition with high levels of recalled frequency of interaction and one condition with low levels of recalled frequency of interaction. An a-priori power-analysis (G*Power [57]) for 1-β = .80, α = .05 and *f*^*2*^ = 0.03 (small effect assumed) for two groups in an ANCOVA with one covariate (digitalization of interaction) indicated an ideal sample size of *N* = 256. Preregistered inclusion criteria were (1) having work experience of at least three years, (2) being employed to the extent of at least 50%, (3) following the instructions regarding frequency of interaction with the leader, (4) passing the attention check, and (5) not being a statistical outlier (for details see below).

We recruited *N* = 320 people to compensate for potential dropouts and exclusions in July 2021 through Prolific Academic. Written informed consent was obtained at the beginning of the study. Two people did not give consent in the beginning of the study, 22 did not have work experience of at least three years, and seven were not employed to the extent of at least 50%. Seventeen further people did not consent to their data being analyzed at the end of the study. Twenty-two did not pass the attention check, and, finally, 65 participants did not follow the instructions regarding the frequency of interaction with the leader. Here, after inducing different memory levels of leader-follower frequency of interaction at the beginning of the survey (high vs. low; for details, see below), participants had to indicate at the end of the survey whether they had answered all questions referring to a past time in which the frequency of interaction with their leader had been high or low. In case they did not follow the instruction (i.e., answered the questions referring to the wrong condition), participants did not pass this check and, thus, were excluded from further analysis. Last, in line with the preregistration, we excluded all outliers on each of the three outcomes using studentized deleted residuals (SDR) from a multiple regression of outcomes on the predictor. Participants with an absolute SDR > |2.59| were excluded, following our standard lab protocol. Thereby, seven participants were excluded.

Accordingly, data from a final sample of *N* = 178 participants were analyzed (*N*_*low-frequency*_ = 78, *N*_*high-frequency*_ = 100, 100 female, 77 male, 1 diverse, *M*_*age*_ = 28.15, *SD* = 6.70). Participants stated to work 38.76 hours per week on average (*SD* = 11.49). The majority were employed in industry and technology (33.1%); other occupational fields were healthcare, social affairs, and education (19.1%), finances, consulting and, insurances (16.3%), and other sectors (31.5%). At the time of the survey, 62.9% of participants had been working for their current employer for 1–5 years.

#### Procedure

Employees were invited to participate in a 10-minute online study on the “perception of the collaboration with leaders”. First, the criteria for participation were checked (i.e., work experience of at least three years, being employed to the extent of at least 50%, and giving consent). Second, to induce different memory levels of (recalled) *frequency of interaction*, all participants were asked to think and write about two times in the past, (1) one in which they had a high and (2) one in which they had a low frequency of interaction with their leader. Following randomized assignment to condition, they were then asked to answer all following questions of the survey referring to the time when they either had rare (low-frequency condition) or frequent (high-frequency condition) contact with their leader.

Then, the task-related leadership outcomes goal clarity, norm clarity, and perceived task responsibility during said (high- or low-frequency) time were assessed, as well as perceived opportunities for work-related information sharing for exploratory purposes (our mediator then tested in Study 4). To ensure the efficacy of our ‘manipulation’, we then implemented a ‘manipulation check’ which participants had to pass to continue with the survey. Here, we asked them which situation they should refer the survey’s questions to (i.e., a time with high vs. low frequency of interaction). Last, several controls were assessed (i.e., valence of interaction; digitalization of interaction between leader and follower; type of digital contact). Finally, participants answered demographic questions (gender, age, working hours, tenure, and their area of business).

#### Measures

Unless indicated otherwise, all items implemented a seven-point scale from 1 =“strongly disagree” to 7 =“strongly agree”.

*Control variable*. *Digitalization of interaction* served as a control variable and was measured with three items (e.g., “My leader and I mostly exchanged questions digitally instead of asking each other in person.”).

*Outcomes*. *Goal clarity* was measured with the same four items as in Studies 1 and 2, however, we slightly changed the items’ phrasing to adjust them to the recalled situation (e.g., “Working with this leader during this time, in my job, most goals were reasonably clear.”). We assessed *norm clarity* with a scale adapted from the ones used in Studies 1 and 2 to exclude items which referred to the whole team, due to the focus of this study on the influence of leaders’ and followers’ dyadic interaction on task-related leadership outcomes. Accordingly, norm clarity was assessed with three items (e.g., “When working on a task with/for my leader during this time, I was always aware of the procedures I was expected to apply.”). Last, we measured perceived *task responsibility* with the same five items as in Studies 1 and 2, with a slightly changed phrasing (adapted to the manipulation; e.g., “At that specific time, working with/for my leader, I felt a great deal of responsibility for the task related to my work.”).

*Additional variables*. We assessed our presumed mediator, *perceived opportunities for work-related information sharing*, as an additional exploratory variable using three items (e.g., “During this time, I experienced that my leader and I had the chance to approach each other regarding work-related issues.”).

### Results

Descriptive statistics, correlations, and Cronbach’s α are displayed in Table 1. Results are shown in Table 6. We conducted three ANCOVAs to test our hypotheses on how high vs. low frequency of interaction (following the procedure in Studies 1–2 including digitalization of interaction, here as control variable) predicts (a) goal clarity, (b) norm clarity, and (c) perceived task responsibility, respectively. Results showed that the two groups differed with respect to their goal clarity, norm clarity, and perceived task responsibility in the expected direction, indicating that the high-frequency group reported more goal clarity, norm clarity, and perceived task responsibility than the low-frequency group (see Table 6). Again, these findings are in line with H1-H3, showing that frequency of interaction predicts better task related leadership outcomes, using a different methodological approach, thereby allowing for greater internal validity than Studies 1–2. The digitalization of interaction also predicted goal clarity, *F*(1, 175) = 15.89, *p* < .001, η_p_^2^ = .083, in that a higher relative amount of digitalization predicted more goal clarity. In contrast, digitalization predicted neither norm clarity, *F*(1, 175) = 0.82, *p* = .366, η_p_^2^ = .005, nor perceived task responsibility, *F*(1, 175) = 3.76, *p* = .054, η_p_^2^ = .021.

**Table 6:** Results of all three ANCOVAs in Study 3 (*N* = 178).

Measure	Low frequency		High frequency		*F*(1, 175)	η_p_^2^
	*M*	*SD*	*M*	*SD*		
Goal Clarity	4.62	1.24	5.38	1.20	31.31	.152***
Norm Clarity	4.99	1.13	5.57	1.08	12.75	.068***
Responsibility	5.46	0.90	5.89	0.81	15.00	.079***

Control variable here was digitalization of interaction. Low frequency = Low frequency of interaction group, High frequency = High frequency of interaction group.

*** *p* < .001.

Note that we also conducted three additional ANOVAs to test for group differences regarding the outcomes *without* controlling for digitalization; again, we found significant differences between the high- and low-frequency groups regarding goal clarity, *F*(1, 176) = 16.98, *p* < .001, η_p_^2^ = .088, norm clarity, *F*(1, 176) = 12.45, *p* = .001, η_p_^2^ = .066, and task responsibility, *F*(1, 176) = 11.15, *p* = .001, η_p_^2^ = .060, demonstrating that the relations between frequency and the outcomes stayed significant also without controlling for digitalization.

#### Additional analyses

Following the general notion of our theoretical model (see Fig 1), we further explored whether the relationships between frequency of interaction and the three outcomes may be mediated by opportunities for work-related information sharing. We conducted mediation analyses (PROCESS, model 4 [61]) with frequency as predictor, digitalization as covariate, perceived opportunities for work-related information sharing as mediator, and the three outcomes, respectively.

**Fig 3:**
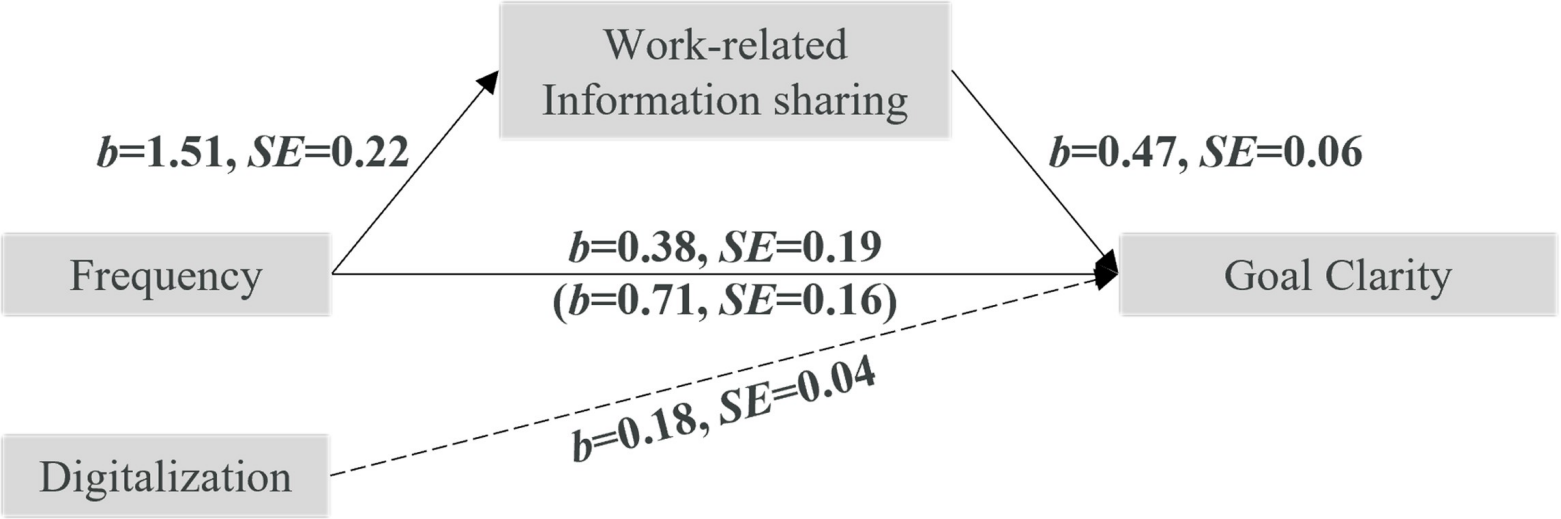
Mediation analysis with outcome goal clarity. Estimates in bold are significant, dashed line represents control variable.

A total effect of frequency on *goal clarity* was observed, *b* = 1.10, *SE* = 0.20, *p* < .001. After entering the mediator into the model, high (vs. low) frequency predicted more opportunities for work-related information sharing (mediator), *b* = 1.51, *SE* = 0.22, *p* < .001, which in turn predicted greater goal clarity, *b* = 0.47, *SE* = 0.06, *p* < .001. The indirect effect was significant; *b* = 0.71, *SE* = 0.16, 95%-CI [0.435, 1.058] (see Fig 3).

**Fig 4:**
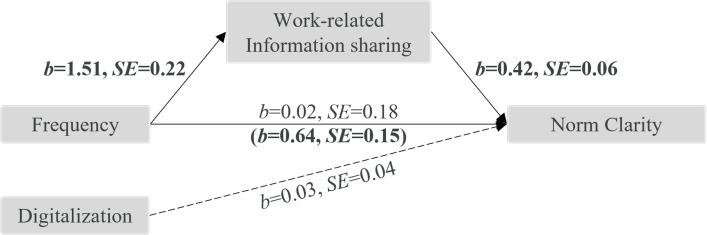
Mediation analysis with outcome norm clarity. Estimates in bold are significant, dashed line represents control variable.

We also found a total effect of frequency on *norm clarity*, *b* = 0.66, *SE* = 0.18, *p* = .001. High (vs. low) frequency once again predicted more opportunities for work-related information sharing (mediator), *b* = 1.51, *SE* = 0.22, *p* < .001, which was linked to greater norm clarity, *b* = 0.42, *SE* = 0.06, *p* < .001, indirect effect *b* = 0.64, *SE* = 0.15, 95%-CI[0.372, 0.949] (see Fig 4).

**Fig 5:**
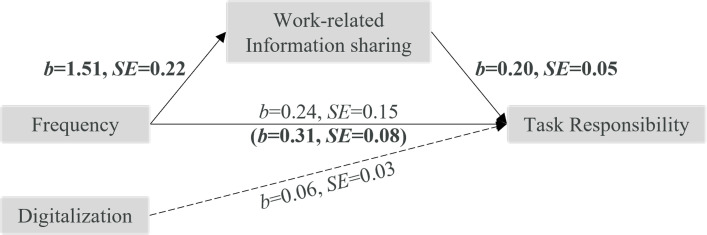
Mediation analysis with outcome perceived task responsibility. Estimates in bold are significant, dashed line represents control variable.

The total effect of frequency on perceived *task responsibility* was significant, *b* = 0.54, *SE* = 0.14, *p* < .001. High (vs. low) frequency again yielded more opportunities for work-related information sharing, *b* = 1.51, *SE* = 0.22, *p* < .001, which in turn predicted higher task responsibility, *b* = 0.20, *SE* = 0.05, *p* < .001, indirect effect *b* = 0.31, *SE* = 0.08, 95%-CI [0.169, 0.473] (see Fig 5).

In sum, these exploratory results suggested that all three relations between frequency and the outcomes may be mediated by more perceived opportunities for work-related information sharing, in that a higher frequency predicted more such opportunities, which in turn were linked to a greater respective outcome.

### Discussion

Study 3 demonstrated that high (vs. low) frequency of interaction (controlling for digitalization of interaction) predicts better leadership outcomes in terms of more follower goal clarity, norm clarity, and perceived task responsibility in the given (recalled) situation. Accordingly, our results support H1, H2, and H3 under more controlled conditions via a quasi-experimental design.

In contrast to Studies 1 and 2, digitalization correlated with goal clarity (but not with the other two outcomes), which thereby partially supports Hypothesis 5. However, given that this is the only case in which we found this relation and that the bivariate correlation between both variables was not significant (but only the correlation in the ANCOVA controlling for the recalled situation), we consider it as an artifact of the analysis rather than as a valid finding.

Interestingly, our additional analyses yielded exploratory support for the general idea that the relationship between higher frequency and better leadership outcomes may be mediated by more perceived opportunities for work-related information sharing between leader and follower (in line with H4). It should, however, be noted that this hypothesis was not preregistered for this study and that the relation between opportunities for work-related information sharing and the outcomes was only tested correlationally. To overcome the downsides of correlational mediation analyses [21], Study 4 followed a causal chain logic—inducing different levels of the *mediator*. We used a quasi-experimental design to induce high (vs. low) levels of perceived opportunities for work-related information sharing (again via recall of specific situations at work) and then assessed the effects on the three main leadership outcomes.

## Study 4: Supporting the process via causal chain

In Study 4, we investigated whether high (vs. low) levels of perceived opportunities for work-related information sharing between leader and follower (our mediator in the overall model) predict better task-related leadership outcomes. Specifically, we hypothesized that high (vs. low) levels of perceived opportunities for work-related information sharing predict higher follower (a) goal clarity, (b) norm clarity, and (c) perceived task responsibility (Hypothesis 4). We induced different levels of (recalled) work-related information sharing with a similar approach as we did with the frequency of interaction in Study 3; we once again assessed our three task-related leadership outcomes goal clarity, norm clarity, and perceived task responsibility during said time. We further measured frequency and digitalization of interaction between leader and follower as exploratory variables.

### Method

#### Transparency and openness

Approval for this study was obtained by the Ethics Committee of the Leibniz-Institut für Wissensmedien under ‘LEK 2021/145’. We describe the sampling plan, data exclusions, and all measures in the study (see table in S8 Table for a full list of measures and table in S9 Table for a comprehensive list of items). The data and research materials of Study 4 are available at https://doi.org/10.23668/psycharchives.12200, the associated analysis code is available at https://doi.org/10.23668/psycharchives.12201. Data were analyzed using SPSS, version 25. The study, including its hypotheses and analyses, was preregistered (https://aspredicted.org/ea3aq.pdf).

#### Design and participants

This study incorporated a quasi-experimental design similar to Study 3. Following a causal chain logic (manipulating the mediator of our model [21]), however, instead of implementing frequency of interaction as dependent variable, we here investigated whether perceived opportunities of work-related information sharing (the mediator in Study 3) predict more follower goal clarity, norm clarity, and perceived task responsibility. The approach was identical to Study 3: by asking participants to remember a time when they perceived many (vs. few) opportunities to share work-related information with their leader, we implemented two between-participants conditions (i.e., high work-related information sharing vs. low work-related information sharing). An a-priori power analysis (G*Power [57]) for 1-β = .80, α = .05, and *f*^*2*^ = 0.03 (small effect assumed) with two groups (high vs. low level of opportunities for information sharing) in an ANOVA indicated an ideal sample size of *N* = 260. Preregistered inclusion criteria were (1) work experience of at least three years, (2) being (or having been) employed to the extent of at least 50%, (3) being at least 20 years old, (4) following the instructions regarding opportunities for work-related information sharing (two ‘manipulation checks’), (5) passing the ‘attention check’, and (6) not being a statistical outlier (for details see below).

We recruited *N* = 475 participants via Clickworker. Informed consent was obtained in written form at the beginning of the study. Thirty-one people did not give consent in the beginning, four did not have work experience of at least three years, and four further participants had not been employed to the extent of at least 50%. Forty-five participants did not reach the end of the survey. Of the remaining 391 participants, 114 did not pass the ‘manipulation’ and/or the ‘attention’ checks, and seven withdrew their data after debriefing. Last, we once again excluded all outliers on each of the three outcomes using studentized deleted residuals (an absolute SDR > |2.59|) from a multiple regression of the respective outcome on the predictor. Thereby, eight participants were excluded.

We analyzed data from a final sample of *N* = 261 (*N*_*low-info-sharing*_ = 137, *N*_*high-info-sharing*_ = 124, 131 female, 127 male, 2 diverse, 1 not indicated, *M*_*age*_ = 39.21, *SD* = 11.24). Participants worked on average 39.21 hours per week (*SD* = 11.24). They indicated being employed in industry and technology (26.4%), healthcare, social affairs, and education (26.1%), finances, consulting, and insurances (11.5%), and other sectors (36.0%). At the time of the survey, 49.8% of participants had been working for their current employer for 1–5 years.

#### Procedure

We invited employees to participate in a 10-minute online study on “the perception of the collaboration with leaders”. At the beginning of the study, the inclusion criteria were assessed. If the participants did not meet one of the criteria, they were automatically excluded from further participation. Subsequently, we induced high (vs. low) memory levels of *perceived opportunities for work-related information sharing* via recall. Participants thought about a time when, in all conversations with their leader, they had talked (1) a lot and (2) very little about work-related issues. To recall these situations more vividly, they wrote down a few notes regarding the topics talked about in these conversations for both situations, respectively (in a counterbalanced order, i.e., the low-information-sharing group first wrote about a time with high information sharing, and then low information sharing; vice versa for the high-information-sharing group). Afterwards, they were asked to answer all following questions with reference to the time, depending on which condition they had been randomly assigned to, when they either talked a lot (high-information-sharing) or very little (low-information-sharing) about work-related issues in all their conversations with their leader. We then implemented a *first* ‘manipulation check’ that participants had to pass to continue survey. Here, we asked them which situation they should refer the survey’s questions to. Afterwards, the three task-related leadership outcomes *goal clarity*, *norm clarity*, and *perceived task responsibility* in that specific situation at work were assessed, followed by the two exploratory variables frequency and digitalization of interaction.

Next, participants had to answer a second manipulation check (they were again asked which situation they had referred the survey’s questions to, i.e., the high or low information sharing situation). For this *second* ‘manipulation check’, we added two more answer options (“both” and “I don’t know anymore”) to reduce the possibility of answering the item correctly by chance.

Subsequently, an ‘attention check’ followed. In case of a failed manipulation and/or attention check, participants were excluded from further participation immediately. Last, we assessed the demographic variables gender, age, weekly working hours, organizational tenure, own leadership position, and working sector.

#### Measures

Unless indicated otherwise, all items implemented a seven-point scale from 1 = “strongly disagree” to 7 = “strongly agree”.

*Outcomes*. *Goal clarity*, *norm clarity*, and *perceived task responsibility* were measured with the same scales as in Study 3.

*Additional measures*. For exploratory purposes, we also assessed *frequency* and *digitalization of interaction* using the same scales as in Study 3.

### Results

Descriptive statistics, correlations, and Cronbach’s α are displayed in Table 1. Results are shown in Table 7. To test H4, we first conducted a one-way MANOVA which showed a significant difference between the two groups of high- vs. low-information-sharing on the three outcomes, *F*(3, 257) = 14.11, *p* < .001, partial η^2^ = .141, Wilk’s Λ = .859.

**Table 7:** Results of all three ANOVAs in Study 4 (*N* = 261).

Measure	Low sharing		High sharing		*F*(1, 259)	η_p_^2^
	*M*	*SD*	*M*	*SD*		
Goal Clarity	4.36	1.52	5.36	1.10	36.90	.125***
Norm Clarity	5.05	1.38	5.57	1.01	11.94	.044**
Responsibility	5.43	1.07	5.88	0.83	14.24	.052***

Low sharing = Low work-related information sharing group, High sharing = High work-related information sharing group.

** *p* < .01.

*** *p* < .001.

Subsequently, we conducted a separate ANOVA for each of the outcome variables, respectively. We found a significant difference between the two groups for goal clarity, norm clarity, and perceived task responsibility (see Table 7). For each outcome, the high-information-sharing group reported higher values on the outcome compared to the low-information-sharing group (and thus, indirectly support the second path captured in H4). Accordingly, these results support the exploratory mediation findings of Study 3 and the general idea that work-related information sharing plays a role in bringing about the beneficial effects on the three leadership outcomes.

### Discussion

In Study 4, following a causal chain logic [21], we compared different levels of opportunities for leader-follower work-related information sharing, our mediator in the overall model (see Fig 1). Results showed that high vs. low levels of perceived opportunities for work-related information sharing indeed predicted more follower goal clarity, norm clarity, and perceived task responsibility. Taken together with the results of Study 3, these findings, therefore, follow a causal chain logic, showing that the effect of frequency on the task-related outcomes is mediated by work-related information sharing. Accordingly, the findings of Study 4 alone indirectly also support Hypothesis 4 and shed light on the mechanism that contributes to bringing about these positive effects on leadership outcomes. While Studies 3 and 4 follow a causal chain logic, the quasi-experimental procedure employed here does not allow to draw conclusions about causality. Combined with the results of the longitudinal Study 2, the current set of studies at least suggest that frequency of interaction affects the three considered outcomes, potentially via work-related information sharing.

## General discussion

Communicating goals, norms, and task responsibilities as one major aspect of successful leadership has become more challenging between leaders and followers, often with increasing distance between the two. This development gives rise to the question of how leaders can still make the relevant goals, norms, and responsibilities clear to followers. Targeting a concrete leadership context—namely, the *frequency* with which followers interact with their leaders—we investigated a possible means to contribute to good task-related leadership outcomes. Specifically, we tested the hypotheses that the frequency and digitalization of interaction between followers and their leaders would predict better (or worse, respectively) task-related leadership outcomes (i.e., goal clarity, norm clarity, and perceived task responsibility among followers); the former as a result of perceiving more opportunities to share work-related information.

Four studies examined these predictions across organizational contexts and using a variety of methodological approaches. In a first step, we tested our predictions cross-sectionally (Study 1) to provide first evidence for our hypotheses. However, this design precluded any conclusions about the direction of the relations found. Study 2 then tested the relations over a time lag of six months (Study 2). As a next step, we compared different levels of frequency of interaction (high vs. low) in a third study (Study 3) to gain quasi-experimental insight into the predicted relations. Study 3 also yielded exploratory evidence in line with the general idea that the relations between frequency and better task-related leadership outcomes are mediated by opportunities for work-related information sharing. Building upon these significant (but not preregistered) mediations for all three outcomes, we conducted a follow-up study (Study 4), in which we compared different (high vs. low) levels of perceived opportunities for work-related information sharing between leader and follower. Here, we found that high (vs. low) levels of (the mediator) opportunities for work-related information sharing did predict more goal clarity, norm clarity, and task responsibility among followers. In sum, these findings support our argument that frequent interactions between leader and follower yield better task-related leadership outcomes due to greater task-related information sharing opportunities.

### Strengths and limitations

Notably, we (successfully) implemented a quasi-experimental approach of ‘inducing’ different memory levels of frequency of interaction. Participants were instructed to think about a time with their leader in which they had interacted to a different extent or shared work-related information to a different extent, respectively, in Studies 3 and 4. This approach allows comparing similar people, even though it is not strictly experimental. However, it adds a different method to the cross-sectional and longitudinal survey approach. Given that the results converge across studies, we overall provide strong evidence for our predictions.

As a limitation, all studies relied on followers’ self-report regarding task-related leadership outcomes and relied on one data source. Extending these via leaders’ perspective or objective measures would be desirable in the future. As a major strength, the studies included followers across organizational fields, supporting our findings’ generalizability. Moreover, we targeted frequency of interaction as a rather objective, though self-reported, predictor that more closely captures what leaders *do* (i.e., how often they are in touch with their followers), rather than how followers subjectively perceive their leaders (e.g., how person-oriented leaders may speak to them). A further major strength of the studies lies in the different approaches we took in order to test our predictions (cross-sectional, longitudinal, and quasi-experimental), thereby strengthening the validity of our findings.

Studies 1–3 supported Hypotheses 1, 2, and 3 (though Study 1 did not support H3 for perceived task responsibility). The more frequent the interaction was, the better the task-related leadership outcomes. Contrary to Hypothesis 5, we found no relations between *digitalization* and the leadership outcomes that go beyond those of frequency (except for goal clarity in Study 3)—which, overall, supports the idea that the effects of leader-follower interaction on task-related leadership outcomes depend on the *frequency* rather than the mode of interaction.

Regarding the lack of effects of digitalization, however, we cannot rule out the possibility that followers and leaders in our studies were already *accustomed* to communicating digitally with each other. While having more digital contact with their leader may affect outcomes early on when establishing a working relationship between leader and follower, the mode may no longer be as important later. Furthermore, in Studies 1 and 2, we did not distinguish between different *forms* of digital contact. It is possible that different forms of digital contact may produce different effects, for instance due to the richness of the information transferred between email as compared to video chat [54, 62]. For this reason, we added the type of digital medium mainly used when communicating digitally as an exploratory variable in Study 3. The type of digital medium neither had a main effect on any outcome, nor did it moderate the relation between digitalization and the outcomes, as we explored in additional analyses. Hence, the type of digital medium does not seem to matter in the relation between digitalization and task-related leadership outcomes.

### Theoretical implications

The findings are relevant to leadership research. Prior approaches discussed the role of increasing *distance* between leaders and followers on different levels [11]. Having digitalized contact can reflect an aspect of physical distance (e.g., collaborating digitally from different geographical locations), but it seems to be an aspect that may not be too relevant regarding leadership outcomes (see our Results). The frequency of interaction, which was the focus of the present research, has been considered a separate aspect of leader-follower distance [11]. Indeed, the present findings highlight that the latter plays a crucial role in predicting task-related leadership outcomes.

From a theoretical perspective, the question of *how* exactly the frequency of interaction—that is, leaders and followers often being in touch with each other—may shape subsequent outcomes was not directly addressed in prior work. In the present research, we sought to address this gap and suggested that via more frequent contact with their followers, leaders may have more chances to *exchange* task-relevant information with each other (e.g., instructions, background information, or feedback [63, 64]); vice versa, doing so may give followers more (perceived) opportunities to contribute their own perspective on task matters or ask questions to their leader. As our results show, both processes likely contribute to greater clarity among followers regarding the goals to pursue, the norms to keep in mind, and the responsibilities to “take care of” as followers. Furthermore, more frequent contact with their followers may enable leaders to better act as role models. Consequently, their followers can infer the relevant goals, norms, and task responsibilities from their leader’s behavior [38]. In line with our predictions, the present findings indicate that sharing work-related information may indeed be a process by which more frequent interactions contribute to better task-related leadership outcomes.

Besides task-related information, it is also possible that leaders and followers use more frequent contact as an opportunity to share *personal* information with each other—an aspect that may contribute to better relationship building and trust [64, 65]. Future research should aim at targeting the mechanisms involved herein and could also consider taking leaders’ perspective into account (e.g., leaders’ clarity of followers’ task-related progress and needs).

But will more frequent contact always contribute to better outcomes in leadership? One may well assume situations in which either party feels that there is *too frequent* contact; leaders may use the opportunity for frequent meetings to convey all their anger about lack of progress or followers may feel controlled or interrupted by a very high frequency of interaction with their leader. Though this was not the focus of the present work, there are certainly limitations to the benefits of frequent contact; for instance, with increasing task expertise and decreasing insecurity, followers may profit less from frequent contact with their leader. This is in line with path-goal theory [66, 67], which states that if goals and paths to goals are already clear, further efforts by the leader to clarify paths and goals may be seen as controlling behavior and interpreted negatively. To take these possibilities into account, we included followers’ perception of the *appropriateness* (Study 1) and *valence* (Studies 1 & 3) of the interactions with their leaders as potential moderator variables in exploratory analyses. Results suggested that most relations seem to be independent of followers’ perceived valence and appropriateness of the interaction (see text in S2 Text and text in S5 Text). Accordingly, in these exploratory analyses, the great majority of relations did not seem to depend on whether followers perceived the interaction as positively (or negatively) valenced or appropriate. This speaks in favor of a relatively robust relation between frequency and leadership outcomes. Still, investigating potential boundary conditions remains an interesting next step for future research.

### Practical implications

The findings are also interesting from a *practical* perspective. In times of increasing distance between leaders and followers due to digitalization, more flexible working arrangements, and the COVID-19 pandemic, many insecurities regarding effective leadership may arise. Our work adds to this line of research by showing that the frequency (rather than the mode of interaction) contributes to leadership outcomes for followers—which is good news in terms of flexible work models. Staying in (frequent) touch with followers may be one relatively easy, practical solution for leaders to ensure their followers’ clarity of goals, norms, and responsibilities, even when ‘traditional’ working conditions and contexts keep changing.

To conclude, leaders and followers nowadays communicate more and more over distance. While digitalized (as compared to face-to-face) contact may have its up- and downsides, the present work suggests that for the clear communication of goals, norms, and tasks between followers and their leaders, an important aspect of communication lies in the *frequency* (rather than the mode) of interaction. Accordingly, leaders may still practice effective leadership despite a potentially greater distance between them and their followers if they manage to stay in touch with each other.

## Supporting information

S1 Table Deviations from preregistrations. (DOCX)

S2 Table Correlations (Cronbach’s alphas in brackets) of all variables in Study 1 (*N* = 200). (DOCX)

S3 Table Item labels of Study 1. (DOCX)

S4 Table Correlations (Cronbach’s alphas in brackets) of all variables initially intended to test in Study 2 (*N* = 305). (DOCX)

S5 Table Item labels of Study 2. (DOCX)

S6 Table Correlations (Cronbach’s alphas in brackets) of all variables in Study 3 (*N* = 178). (DOCX)

S7 Table Item labels of Study 3. (DOCX)

S8 Table Correlations (Cronbach’s alphas in brackets) of all variables in Study 4 (*N* = 261). (DOCX)

S9 Table Item labels of Study 4. (DOCX)

S1 Text Research materials Study 1. (DOCX)

S2 Text Additional analyses Study 1. (DOCX)

S3 Text Research materials Study 2. (DOCX)

S4 Text Research materials Study 3. (DOCX)

S5 Text Additional analyses Study 3. (DOCX)

S6 Text Research materials Study 4. (DOCX)
