# Supplementary material for: Let’s stay in touch: Frequency (but not mode) of interaction between leaders and followers predicts better leadership outcomes
Source: PLoS One. 2022 Dec 22;17(12):e0279176. doi: 10.1371/journal.pone.0279176 (PMC9778566; doi:10.1371/journal.pone.0279176)
Supplement: S1 Table — (DOCX) [file pone.0279176.s001.docx]

**S1 Table. Deviations from preregistrations.**

| **Study** | **Paper vs. Preregistration** |
| --- | --- |
| **Study 1** | ***Variable labels****:*  *Paper*: predictor is labeled frequency of interaction *Preregistration*: predictor is labeled interaction frequency  *Paper*: variable is labeled norm clarity  *Preregistration*: variable is labeled norms clarity  ***Hypotheses***: *Paper*: order of H1 and H3 are reversed (vs. Preregistration) *Paper*: H5 predicts an effect of digitalization on the outcomes; this hypothesis was not included in the preregistration (in which we intended to use digitalization only as control variable, see below)  *Paper:* “Followers” were initially named “subordinates” in the preregistration  ***Analyses***: *Paper*: digitalization is labeled predictor and treated as such *Preregistration*: digitalization is labeled control variable |
| **Study 2** | ***Variable labels:*** *Paper*: variable is labeled goal clarity  *Preregistration*: variable is labeled clarity of goals   ***Analyses and hypotheses***: *Paper*: tests the effects of frequency and digitalization on leadership outcomes *Preregistration*: original aim was to test whether frequency moderates the relationship between organizational guidance (i.e., transformational leadership, clarity of goals, norm clarity, and subordinates’ perceived task responsibility) and leadership outcomes in subordinates (i.e., autonomous motivation and perceived leadership effectiveness). |
| **Study 3** | ***Variable labels:***  *Paper:* variable is labeled opportunities for work-related information sharing  *Preregistration:* variable is labeled work-related information sharing |
| **Study 4** | ***Variable labels:***  *Paper:* condition is labeled “high/low opportunities work-related information sharing”  *Preregistration:* condition is labeled “high/low work-related information sharing”  ***Hypotheses:***  *Paper*: H4 predicts opportunities for work-related information sharing as mediator of the relations between frequency and the outcomes  *Preregistration*: the specific hypothesis preregistered here follows the quasi-experimental design logic and refers to the *second* path of the mediation (mediator => outcome); accordingly, it predicts that high (vs. low) work-related information sharing between leader and follower leads to (a) more follower goal clarity, (b) more follower norm clarity, and (c) more follower perceived task responsibility. |
