# Supplementary material for: Let’s stay in touch: Frequency (but not mode) of interaction between leaders and followers predicts better leadership outcomes
Source: PLoS One. 2022 Dec 22;17(12):e0279176. doi: 10.1371/journal.pone.0279176 (PMC9778566; doi:10.1371/journal.pone.0279176)
Supplement: S2 Table — (DOCX) [file pone.0279176.s002.docx]

| Variables | *M* | *SD* | (1) | (2) | (3) | (4) | (5) | (6) | (7) | (8) | (9) | (10) | (11) |
| --- | --- | --- | --- | --- | --- | --- | --- | --- | --- | --- | --- | --- | --- |
| (1) Freq | 4.40 | 1.16 | (.86) |  |  |  |  |  |  |  |  |  |  |
| (2) Dig | 82.00 | 26.14 | -.15^*^ | (-) |  |  |  |  |  |  |  |  |  |
| (3) Goal | 4.72 | 1.41 | .25^***^ | -.01 | (.84) |  |  |  |  |  |  |  |  |
| (4) Norm | 4.64 | 0.87 | .17^*^ | .03 | .51^***^ | (.75) |  |  |  |  |  |  |  |
| (5) Resp | 6.06 | 0.68 | -.02 | .05 | .11 | .15^*^ | (.62) |  |  |  |  |  |  |
| (6) Lresp | 4.51 | 1.43 | .36^***^ | .02 | .31^***^ | .19^**^ | -.02 | (.92) |  |  |  |  |  |
| (7) Trust | 5.48 | 1.35 | .41^***^ | -.02 | .32^***^ | .18^*^ | .02 | .74^***^ | (.88) |  |  |  |  |
| (8) LMX | 3.64 | 0.76 | .44^***^ | .11 | .41^***^ | .33^***^ | .08 | .77^***^ | .76^***^ | (.91) |  |  |  |
| (9) Ident | 4.73 | 1.23 | .11 | -.03 | .13 | .22^**^ | .30^***^ | .11 | .12 | .19^**^ | (.87) |  |  |
| (10) Val | 4.04 | 0.95 | .26^***^ | .00 | .25^***^ | .16^*^ | -.08 | .62^***^ | .65^***^ | .65^***^ | .02 | (.80) |  |
| (11) Appr | 4.21 | 1.12 | .42^***^ | .05 | .35^***^ | .23^**^ | .03 | .46^***^ | .45^***^ | .54^***^ | .12 | .48^***^ | (.63) |

**S3 Table. Correlations (Cronbach’s alphas in brackets) of all variables in Study 1 (*N* = 200)**

Freq = Frequency of interaction, Dig = Digitalization of interaction, Goal = Goal clarity, Norm = Norm clarity, Resp = Task Responsibility, Lresp = Perceived leader responsibility, Ident = Organizational identification, Val = Valence of interaction, Appr = Appropriateness of interaction. For (10) Val and (11) Appr we report Pearson’s r instead of Cronbach’s alpha, due to both scales consisting of two items.

^*^ *p* < .05.

^**^ *p* < .01.

^***^ *p* < .001.
