# Supplementary material for: Let’s stay in touch: Frequency (but not mode) of interaction between leaders and followers predicts better leadership outcomes
Source: PLoS One. 2022 Dec 22;17(12):e0279176. doi: 10.1371/journal.pone.0279176 (PMC9778566; doi:10.1371/journal.pone.0279176)
Supplement: S3 Table — (DOCX) [file pone.0279176.s003.docx]

**S4 Table. Item labels of Study 1.**

| **Abbreviation** | **Item** | **Item Label** | **Scale** |
| --- | --- | --- | --- |
| Freq1 | Frequency1 | *How frequently did you interact with your leader at work?* | **Frequency of interaction** |
| Freq2 | Frequency2 | *How frequently did your leader initiate work-related interaction with you?* |  |
| Freq3 | Frequency3 | *How frequently did you initiate work-related interaction with your leader?* |  |
| Freq4 | Frequency4 | *How frequently did you interact with your leader informally or socially at work?* |  |
| Dig | Digitalization | *What percentage of your weekly working time did you work away from your office (e.g., home-office)?* | **Digitalization of interaction** |
| Goal1 | Goal clarity1 | *In my job, in general, goals are poorly described.* | **Goal clarity** |
| Goal2 | Goal clarity2 | *In my job, most goals are vague.* |  |
| Goal3 | Goal clarity3 | *In my job, most goals are reasonably clear.* |  |
| Goal4 | Goal clarity4 | *In my job, the tasks that my leader gives me are linked to long range goals.* |  |
| Norm1 | Norm clarity1 | *In my team, we agree about how things should be done.* | **Norm clarity** |
| Norm2 | Norm clarity2 | *In my team, we apply the same approach when performing the same work task.* |  |
| Norm3 | Norm clarity3 | *In my team, we are expected to apply the same procedure when doing the same task.* |  |
| Norm4 | Norm clarity4 | *In my team, procedures and rules how to do things frequently change.* |  |
| Norm5 | Norm clarity5 | *When working on a task, I know the expectations regarding the strategies I should apply.* |  |
| Norm6 | Norm clarity6 | *When working on a task, rules and norms regarding my task are clear to me.* |  |
| Norm7 | Norm clarity7 | *When working on a task, I always follow the procedures that I am expected to apply.* |  |
| Norm8 | Norm clarity8 | *When working on a task, we, as a team, use the procedures preferred by our leader.* |  |
| Resp1 | Responsibility1 | *I feel a great deal of responsibility for the tasks related to my work.* | **Perceived task responsibility** |
| Resp2 | Responsibility2 | *I see it as a duty to make my job as good as possible.* |  |
| Resp3 | Responsibility3 | *I am always thinking about how my actions will affect the organization’s goals.* |  |
| Resp4 | Responsibility4 | *I feel personally accountable if something goes wrong at work.* |  |
| Resp5 | Responsibility5 | *I am usually concerned about the progress of the projects I am involved in.* |  |
| Val1 | Valence1 | *How did you perceive the contact by itself?* | **Valence of interaction** |
| Val2 | Valence2 | *How did you perceive the atmosphere of the contact?* |  |
| Appr1 | Appropriateness1 | *How did you perceive the frequency of contact?* | **Appropriateness of interaction** |
| Appr2 | Appropriateness2 | *How did you perceive the intensity (e.g., duration, detailedness) of contact?* |  |
