# Supplementary material for: Let’s stay in touch: Frequency (but not mode) of interaction between leaders and followers predicts better leadership outcomes
Source: PLoS One. 2022 Dec 22;17(12):e0279176. doi: 10.1371/journal.pone.0279176 (PMC9778566; doi:10.1371/journal.pone.0279176)
Supplement: S4 Table — (DOCX) [file pone.0279176.s004.docx]

**S7 Table. Correlations (Cronbach’s alphas in brackets) of all variables initially intended to test in Study 2 (N = 305)**

| Variable | *M* | *SD* | (1) | (2) | (3) | (4) | (5) | (6) | (7) | (8) | (9) | (10) | (11) | (12) | (13) | (14) | (15) | (16) |  |
| --- | --- | --- | --- | --- | --- | --- | --- | --- | --- | --- | --- | --- | --- | --- | --- | --- | --- | --- | --- |
| Time 1 |  |  |  |  |  |  |  |  |  |  |  |  |  |  |  |  |  |  |  |
| (1) Freq | 5.31 | 1.24 | (.79) |  |  |  |  |  |  |  |  |  |  |  |  |  |  |  |  |
| (2) Dig | 3.24 | 1.81 | -.39^***^ | (.67) |  |  |  |  |  |  |  |  |  |  |  |  |  |  |  |
| (3) Goal | 4.72 | 1.36 | .29^***^ | -.08 | (.86) |  |  |  |  |  |  |  |  |  |  |  |  |  |  |
| (4) Norm | 5.15 | 0.83 | .29^***^ | -.14^**^ | .58^***^ | (.81) |  |  |  |  |  |  |  |  |  |  |  |  |  |
| (5) Resp | 5.44 | 1.01 | .22^***^ | .02 | .36^***^ | .35^***^ | (.81) |  |  |  |  |  |  |  |  |  |  |  |  |
| (6) TFL | 3.66 | 0.99 | .52^***^ | -.10^*^ | .59^***^ | .47^***^ | .35^***^ | (.94) |  |  |  |  |  |  |  |  |  |  |  |
| (7) Amot | 3.65 | 0.72 | .21^***^ | -.00 | .29^***^ | .30^***^ | .55^***^ | .40^***^ | (.91) |  |  |  |  |  |  |  |  |  |  |
| (8) Effect | 5.13 | 1.64 | .61^***^ | -.12^*^ | .51^***^ | .39^***^ | .27^***^ | .86^***^ | .27^***^ | (.97) |  |  |  |  |  |  |  |  |  |
| Time 2 |  |  |  |  |  |  |  |  |  |  |  |  |  |  |  |  |  |  |  |
| (9) Freq | 6.06 | 0.96 | .71^***^ | -.36^***^ | .22^***^ | .30^***^ | .26^***^ | .45^***^ | .24^***^ | .47^***^ | (.71) |  |  |  |  |  |  |  |  |
| (10) Dig | 2.99 | 1.48 | -.31^***^ | .70^***^ | -.03 | -.14^*^ | -.02 | -.07 | .03 | -.08 | -.36^***^ | (.70) |  |  |  |  |  |  |  |
| (11) Goal | 4.86 | 1.33 | .32^***^ | -.10 | .66^***^ | .50^***^ | .30^***^ | .48^***^ | .28^***^ | .43^***^ | .24^***^ | -.02 | (.87) |  |  |  |  |  |  |
| (12) Norm | 5.17 | 0.86 | .34^***^ | -.17^**^ | .41^***^ | .64^***^ | .24^***^ | .42^***^ | .27^***^ | .38^***^ | .37^***^ | -.07 | .61^***^ | (.83) |  |  |  |  |  |
| (13) Resp | 5.47 | 0.98 | .30^***^ | -.02 | .27^***^ | .31^***^ | .69^***^ | .29^***^ | .49^***^ | .24^***^ | .27^***^ | .08 | .36^***^ | .40^***^ | (.82) |  |  |  |  |
| (14) TFL | 3.72 | 0.99 | .40^***^ | -.05 | .47^***^ | .37^***^ | .32^***^ | .73^***^ | .39^***^ | .66^***^ | .47^***^ | .03 | .55^***^ | .52^***^ | .39^***^ | (.95) |  |  |  |
| (15) Amot | 3.58 | 0.74 | .24^***^ | -.04 | .25^***^ | .24^***^ | .42^***^ | .33^***^ | .78^***^ | .21^***^ | .25^***^ | .05 | .31^***^ | .32^***^ | .50^***^ | .40^***^ | (.92) |  |  |
| (16) Effect | 5.20 | 1.56 | .45^***^ | -.09 | .42^***^ | .33^***^ | .21^***^ | .70^***^ | .28^***^ | .72^***^ | .53^***^ | -.05 | .49^***^ | .49^***^ | .28^***^ | .87^***^ | .27^***^ | (.97) |  |

Resp = followers’ perceived task responsibility; TFL = Transformational Leadership Behavior; Amot = Autonomous motivation; effect = Leader effectiveness

*^*^ p* < .05.

^**^ *p* < .01.

^***^ *p* < .001.
