# Supplementary material for: Let’s stay in touch: Frequency (but not mode) of interaction between leaders and followers predicts better leadership outcomes
Source: PLoS One. 2022 Dec 22;17(12):e0279176. doi: 10.1371/journal.pone.0279176 (PMC9778566; doi:10.1371/journal.pone.0279176)
Supplement: S6 Table — (DOCX) [file pone.0279176.s006.docx]

| Variables | *M* | *SD* | (1) | (2) | (3) | (4) | (5) | (6) | (7) | (8) | (9) | (10) | (11) | (12) |
| --- | --- | --- | --- | --- | --- | --- | --- | --- | --- | --- | --- | --- | --- | --- |
| (1) Dig | 4.37 | 1.96 | (.92) |  |  |  |  |  |  |  |  |  |  |  |
| (2) Goal | 5.04 | 1.27 | .12 | (.79) |  |  |  |  |  |  |  |  |  |  |
| (3) Norm | 5.32 | 1.13 | -.05 | .57^***^ | (.74) |  |  |  |  |  |  |  |  |  |
| (4) Resp | 5.70 | 0.87 | .02 | .30^***^ | .42^***^ | (.73) |  |  |  |  |  |  |  |  |
| (5) Work | 5.31 | 1.49 | -.17^*^ | .58^***^ | .55^***^ | .39^***^ | (.91) |  |  |  |  |  |  |  |
| (6) Freq | 4.60 | 1.24 | -.26^***^ | .27^***^ | .36^***^ | .26^**^ | .48^***^ | (.80) |  |  |  |  |  |  |
| (7) Val | 5.06 | 1.48 | -.11 | .60^***^ | .53^***^ | .40^***^ | .70^***^ | .44^***^ | (.91) |  |  |  |  |  |
| (8) LMX | 3.45 | 0.82 | -.08 | .61^***^ | .57^***^ | .35^***^ | .70^***^ | .55^***^ | .81^***^ | (.86) |  |  |  |  |
| (9) Char | 4.68 | 1.44 | .04 | .52^***^ | .52^***^ | .43^***^ | .55^***^ | .34^***^ | .77^***^ | .77^***^ | (.90) |  |  |  |
| (10) Pers | 3.66 | 1.89 | -.06 | .33^***^ | .39^***^ | .38^***^ | .46^***^ | .43^***^ | .59^***^ | .58^***^ | .55^***^ | (.92) |  |  |
| (11) TNorm | 4.80 | 1.13 | .06 | .41^***^ | .45^***^ | .24^**^ | .43^***^ | .42^***^ | .58^***^ | .62^***^ | .58^***^ | .40^***^ | (.63) |  |
| (12) Inter | 5.12 | 0.94 | .24^**^ | .39^***^ | .34^***^ | .27^***^ | .39^***^ | .28^***^ | .43^***^ | .39^***^ | .42^***^ | .27^***^ | .50^***^ | (.71) |

**S10 Table. Correlations (Cronbach’s alphas in brackets) of all variables in Study 3 (N = 178).**

Freq = Frequency of interaction, Dig = Digitalization of interaction, Goal = Goal clarity, Norm = Norm clarity, Resp = Task Responsibility, Val = Valence of interaction. Work = Work-related information sharing, Char = Leader charisma, Pers = Personal information sharing, TNorm = Team norm clarity, Inter = Perceived interactivity of digital medium. For (10) Val we report Pearson’s r instead of Cronbach’s alpha, due to both scales consisting of two items.

*^*^ p* < .05.

^**^ *p* < .01.

^***^ *p* < .001.
