# Supplementary material for: Let’s stay in touch: Frequency (but not mode) of interaction between leaders and followers predicts better leadership outcomes
Source: PLoS One. 2022 Dec 22;17(12):e0279176. doi: 10.1371/journal.pone.0279176 (PMC9778566; doi:10.1371/journal.pone.0279176)
Supplement: S8 Table — (DOCX) [file pone.0279176.s008.docx]

| Variables | *M* | *SD* | (1) | (2) | (3) | (4) | (5) | (6) |
| --- | --- | --- | --- | --- | --- | --- | --- | --- |
| (1) Goal | 4.84 | 1.43 | (.84) |  |  |  |  |  |
| (2) Norm | 5.30 | 1.24 | .63^***^ | (.81) |  |  |  |  |
| (3) Resp | 5.64 | 0.99 | .33^***^ | .43^***^ | (.81) |  |  |  |
| (4) Dig | 3.85 | 1.93 | .03 | -.02 | -.04 | (.89) |  |  |
| (5) Freq | 4.42 | 1.21 | .25^***^ | .25^***^ | .17^**^ | -.34^***^ | (.81) |  |
| (6) Work | 5.37 | 1.22 | .56^***^ | .59^***^ | .43^***^ | -.01 | .37^***^ | (.88) |

**S14 Table. Correlations (Cronbach’s alphas in brackets) of all variables in Study 4 (*N* = 261).**

Goal = Goal clarity, Norm = Norm clarity, Resp = Task Responsibility, Freq = Frequency of interaction, Dig = Digitalization of interaction, Work = Work-related information sharing

*^*^ p* < .05.

^**^ *p* < .01.

^***^ *p* < .001.
