# Supplementary material for: Let’s stay in touch: Frequency (but not mode) of interaction between leaders and followers predicts better leadership outcomes
Source: PLoS One. 2022 Dec 22;17(12):e0279176. doi: 10.1371/journal.pone.0279176 (PMC9778566; doi:10.1371/journal.pone.0279176)
Supplement: S9 Table — (DOCX) [file pone.0279176.s009.docx]

**S15 Table. Item labels of Study 4.**

| **Abbreviation** | **Item** | **Item Label** | **Scale** |
| --- | --- | --- | --- |
| Goal1 | Goal clarity1 | *Working with this leader during this time, in my job, goals were poorly described.* | **Goal clarity** |
| Goal2 | Goal clarity2 | *Working with this leader during this time, in my job, most goals were vague.* |  |
| Goal3 | Goal clarity3 | *Working with this leader during this time, in my job, most goals were reasonably clear.* |  |
| Goal4 | Goal clarity4 | *Working with this leader during this time, in my job, the tasks that my leader gave me were linked to long range goals.* |  |
| Norm1 | Norm clarity1 | *When working on a task with/for my leader during this time, I knew the expectations regarding the strategies I should apply.* | **Norm clarity** |
| Norm2 | Norm clarity2 | *When working on a task with/for my leader during this time, I was always aware of the procedures that I was expected to apply.* |  |
| Norm3 | Norm clarity3 | *When working on a task with/for my leader during this time, I used the procedures preferred by this leader.* |  |
| Resp1 | Responsibility1 | *At that specific time, working for / with my leader, I felt a great deal of responsibility for the tasks related to my work.* | **Perceived task responsibility** |
| Resp2 | Responsibility2 | *At that specific time, working for / with my leader, I saw it as a duty to make my job as good as possible.* |  |
| Resp3 | Responsibility3 | *At that specific time, working for / with my leader, I was always thinking about how my actions will affect the organizations goals.* |  |
| Resp4 | Responsibility4 | *At that specific time, working for / with my leader, I felt personally accountable if something went wrong at work.* |  |
| Resp5 | Responsibility5 | *At that specific time, working for / with my leader, I was usually concerned about the progress of the projects I was involved in.* |  |
| Freq1 | Frequency1 | *How frequently did this leader initiate work-related contact with you?* | **Frequency of interaction** |
| Freq2 | Frequency2 | *How frequently did you initiate work-related contact with this leader?* |  |
| Freq3 | Frequency3 | *How frequently did you interact with this leader at work?* |  |
| Freq4 | Frequency4 | *How frequently did you interact with this leader informally or socially at work?* |  |
| Dig1 | Digitalization1 | *My leader and I mostly exchanged questions digitally instead of asking each other in person.* | **Digitalization of interaction** |
| Dig2 | Digitalization2 | *My leader and I mostly interacted using media (e.g., phone, email) rather than talking face-to-face.* |  |
| Dig3 | Digitalization3 | *To what extent did the contact during that time occur digitally or via telephone, but not face-to-face? Please consider every form of digital contact (e.g., email, video chat, etc.)* **Percent slider** |  |
