# Supplementary material for: Let’s stay in touch: Frequency (but not mode) of interaction between leaders and followers predicts better leadership outcomes
Source: PLoS One. 2022 Dec 22;17(12):e0279176. doi: 10.1371/journal.pone.0279176 (PMC9778566; doi:10.1371/journal.pone.0279176)
Supplement: S1 Text — (DOCX) [file pone.0279176.s010.docx]

**S2 Text. Research materials Study 1.**

*Complete list of variables assessed in this study (in the actual survey order):* Variables in bold are included in the manuscript; variables not in bold were assessed for a different research question not targeted in this manuscript. Abbreviations in brackets are presented in Table 1.

- **Frequency of interaction (Freq)**
- **Digitalization of interaction (Dig)**
- **Perceived task responsibility (Resp)**
- **Goal clarity (Goal)**
- **Norm clarity (Norm)**
- Organizational identification (Ident, 6 items)
- Leader-Member-Exchange (LMX, 7 items; LMX7, Graen & Uhl-Bien, 1995)
- Perceived leader responsibility (LResp, 5 items; Scholl et al., 2017; 2018)
- Leader trustworthiness (Trust, 3 items; Winter et al., 2021)
- Appropriateness of interaction (Appr, 2 items; self-developed)
- Valence of interaction (Val, 2 items; self-developed)
