# Supplementary material for: Let’s stay in touch: Frequency (but not mode) of interaction between leaders and followers predicts better leadership outcomes
Source: PLoS One. 2022 Dec 22;17(12):e0279176. doi: 10.1371/journal.pone.0279176 (PMC9778566; doi:10.1371/journal.pone.0279176)
Supplement: S2 Text — (DOCX) [file pone.0279176.s011.docx]

S5 Text. Additional analyses Study 1.

Exploratory moderation analyses via PROCESS (version 3.5, Hayes, 2018; Model 1) tested whether the relationship between frequency of interaction (entered as predictor) and each of the leadership outcomes (outcomes) is moderated by followers’ perceived valence and appropriateness of the interaction (moderators), respectively.

The *valence of interaction* did moderate the relationship between frequency of interaction and norm clarity (*b* = 0.12, *SE* = 0.06, *p* = .031) In case of a more positive valence of interaction (+1 *SD*), frequency of interaction and norm clarity were positively related (*b* = 0.22, *SE* = 0.08, *p* = .004), whereas they were unrelated in case of a less positive valence of interaction (-1 *SD*, *b* = -0.02, *SE* = 0.08, *p* = .829*)*. However, valence of interaction neither moderated the relationship between frequency and goal clarity (*b* = 0.09, *SE* = 0.09, *p* = .309) nor between frequency and perceived task responsibility (*b* = -0.03, *SE* = 0.04, *p* = .571).

Similarly, the *appropriateness of interaction* neither moderated the relationship of frequency with goal clarity (*b* = 0.00, *SE* = 0.08, *p* = .992), nor norm clarity (*b* = 0.05, *SE* = 0.05, *p* = .328), nor perceived task responsibility (*b* = -0.02, *SE* = 0.04, *p* = .583).

Accordingly, in sum, the relationship between frequency of interaction and the task-related outcomes did (with one exception with a borderline *p*-value) *not* depend on whether followers perceived the interaction as (not) positively valenced or the frequency as (not) appropriate. Due to the exploratory nature of these analyses, these results need to be interpreted with caution.
