# Supplementary material for: Let’s stay in touch: Frequency (but not mode) of interaction between leaders and followers predicts better leadership outcomes
Source: PLoS One. 2022 Dec 22;17(12):e0279176. doi: 10.1371/journal.pone.0279176 (PMC9778566; doi:10.1371/journal.pone.0279176)
Supplement: S3 Text — (DOCX) [file pone.0279176.s012.docx]

**S6 Text. Research Materials Study 2.**

**Original focus of the study**

The initial purpose of the study was to test whether the frequency of interaction between leader and subordinates moderates the relationship between organizational guidance (i.e., transformational leadership, goal clarity, norm clarity, and subordinates’ perceived task responsibility) and leadership outcomes (i.e., autonomous motivation and perceived leadership effectiveness). Unexpectedly high correlations of *transformational leadership* with all other variables, however, precluded us from performing these analyses (see Table 10). For that reason, we decided to continue with another, exploratory approach as presented in the paper that targets the outcomes of frequency of interaction.

*Complete list of variables assessed in this study (in the actual survey order):*

Variables in bold are included in the manuscript; variables in blue and not in bold were assessed for a different research question not targeted in this manuscript. Variables in black and not in bold represent variables related to leadership and were assessed for the initial purpose of the study but are not included in the manuscript.

- Social Identification with the organization (10 items; adapted from Leach et al., 2008; Mael & Ashforth, 1992)
- Excellence norms in the organization (6 items; Scholl et al., 2019)
- Threat/challenge/hindrance appraisal of excellence norms (11 items; Feldman et al., 2004; Tuckey et al., 2015)
- External motivation to perform well (4 items)
- Autonomous motivation (13 items; adapted from Vallerand et al., 1992)
- Social support from colleagues (3 items)
- Transformational leadership (TFL, 7 items; Carless et al., 2000)
- **Goal Clarity (Goal)**
- **Norm clarity (Norm)**
- **Responsibility (Resp)**
- Leader effectiveness (Effect, 4 items; Giessner & van Knippenberg, 2008)
- **Frequency of Interaction (Freq)**
- **Digitalization of Interaction (Dig)**
- Workload (5 items; Specter & Jex, 1998)
- Personal striving for excellence (3 items)
- Self-efficacy (3 items)
- Self-reported work performance (1 item)
- Well-being (5 items; WHO 5, Bech et al., 2003)
- Stress symptoms (14 items; HSCL; Hesbacher et al., 1980)
- Emotional Exhaustion (8 items; Demerouti et al., 2010)
- Number of sick days (1 item)
- Resentment (7 items)
- Trait negative affect (20 items; PANAS, Sonnentag et al., 2008; Watson et al., 1988)
