# Supplementary material for: Let’s stay in touch: Frequency (but not mode) of interaction between leaders and followers predicts better leadership outcomes
Source: PLoS One. 2022 Dec 22;17(12):e0279176. doi: 10.1371/journal.pone.0279176 (PMC9778566; doi:10.1371/journal.pone.0279176)
Supplement: S4 Text — (DOCX) [file pone.0279176.s013.docx]

**S9 Text. Research materials Study 3.**

*Complete list of variables assessed in this study (in the actual survey order):* Variables in bold are included in the manuscript; variables not in bold were assessed for exploratory purposes; variables in blue and not in bold were assessed for a different research question not targeted in this manuscript. Abbreviations in brackets are presented in Table 12.

- **Digitalization of interaction (Dig)**
- **Goal clarity (Goal)**
- **Norm clarity (Norm)**
- **Perceived task responsibility (Resp)**
- **Work-related information sharing (Work)**
- Frequency of interaction (Freq)
- Valence of interaction (Val, 2 items; self-developed)
- Followers’ perceived interactivity of digital medium (Inter, 6 items; self-developed)
- Leader-Member-Exchange (LMX, 6 items; LMX7, adapted from Graen & Uhl-Bien, 1995)
- Perceived leader charisma (Char, 5 items; self-developed)
- Personal information sharing (Pers, 3 items; self-developed)
- Team norm clarity (TNorm, 4 items; self-developed)
