# Supplementary material for: Let’s stay in touch: Frequency (but not mode) of interaction between leaders and followers predicts better leadership outcomes
Source: PLoS One. 2022 Dec 22;17(12):e0279176. doi: 10.1371/journal.pone.0279176 (PMC9778566; doi:10.1371/journal.pone.0279176)
Supplement: S5 Text — (DOCX) [file pone.0279176.s014.docx]

**S12 Text. Additional analyses Study 3.**

As in Study 1, we explored whether the effects of frequency on the outcomes are moderated by followers’ perceived valence of the interaction (model 1, Hayes, 2020). As in Study 1, the valence of interaction moderated the relationship between frequency and norm clarity in case of a more positive valence (+ 1 *SD*, *b* = 0.52, *SE* = 0.23, *p* = .024), whereas frequency and norm clarity were unrelated in case of a less positive valence of interaction (-1 *SD, b* = -0.19, *SE* = 0.22, *p* = .384). However, valence of interaction neither moderated the relationship between frequency and goal clarity (*b* = 0.18, *SE* = 0.11, *p* = .105) nor between frequency and perceived task responsibility (*b* = 0.13, *SE* = 0.09, *p* = .157).

Accordingly, as in Study 1, except for norm clarity, the relations between frequency and the task-related outcomes were not influenced by whether followers perceived the interaction as negatively or positively valenced. Again, since these results are exploratory, they need confirmatory testing.
