# Supplementary material for: Let’s stay in touch: Frequency (but not mode) of interaction between leaders and followers predicts better leadership outcomes
Source: PLoS One. 2022 Dec 22;17(12):e0279176. doi: 10.1371/journal.pone.0279176 (PMC9778566; doi:10.1371/journal.pone.0279176)
Supplement: S6 Text — (DOCX) [file pone.0279176.s015.docx]

**S13 Text. Research materials Study 4.**

*Complete list of variables assessed in this study (in the actual survey order):* Variables in bold are included in the manuscript; variables not in bold were assessed for exploratory purposes. Abbreviations in brackets are presented in Table 14.

- **Goal clarity (Goal)**
- **Norm clarity (Norm)**
- **Perceived task responsibility (Resp)**
- Digitalization of interaction (Dig, 3 items; self-developed
- Frequency of interaction (Freq, 4 items; adapted from McAllister, 1995)
- Work-related information sharing (Work, 3 items; self-developed)
